# The Impact of Risk Factors and Comorbidities on Heart Failure in the Aging Population

**DOI:** 10.3390/ijms27125296

**Published:** 2026-06-11

**Authors:** Ruzzell C. Flores, Fatin Shadab Talukder, Inna Rabinovich-Nikitin

**Affiliations:** 1 Department of Physiology and Pathophysiology, Rady College of Medicine, Max Rady Faculty of Health Sciences, University of Manitoba, Winnipeg, MB R2H2A6, Canada; rflores@sbrc.ca (R.C.F.); 2 The Institute of Cardiovascular Sciences, St. Boniface Hospital Albrechtsen Research Centre, Winnipeg, MB R2H2A6, Canada

**Keywords:** aging, heart failure, obesity, diabetes, hypertension, smoking, sleep apnea

## Abstract

Heart failure (HF) represents a major and escalating public health challenge in the aging population. Older adults with HF frequently present with coexisting conditions such as hypertension, diabetes mellitus and obesity, which substantially influence disease pathophysiology, clinical presentation, therapeutic response and prognosis. The complex interplay between HF risk factors and comorbidities complicates diagnosis, limits the applicability of guideline-directed therapies and contributes to increased symptom burden, recurrent hospitalizations, higher healthcare utilization and reduced quality of life. In this Review, we examine the mechanisms through which common risk factors and comorbidities modify HF progression and outcomes in elderly patients, highlighting their impact on disease severity and treatment effectiveness. We emphasize the need for individualized, patient-centered management strategies that move beyond a single-disease framework and incorporate multidisciplinary care models. Early identification of comorbidities tailored pharmacological and non-pharmacological interventions, and longitudinal monitoring are essential to address the dual burden of HF and multimorbidity. Finally, we discuss current knowledge gaps and future research priorities, including the need to elucidate shared pathophysiological pathways and to develop integrated therapeutic approaches. Advancing our understanding of HF in the context of aging and risk factor/comorbidity is critical for improving clinical outcomes and informing healthcare policy in this growing patient population.

## 1. Introduction

Cardiovascular disease (CVD) is the leading cause of death among individuals aged 65 and older [1]. As risk factors for heart conditions continue to rise within the aged population [2], the incidence of heart failure (HF)—a condition where the heart is unable to meet the demand of the body is also increased [3]. HF is a clinical syndrome caused by structural and/or functional cardiac abnormalities that impair ventricular filling or ejection, resulting in symptoms and/or signs such as dyspnea, fatigue, and pulmonary or systemic congestion [4]. HF is a major and growing public health issue associated with substantial morbidity and mortality [5]. In the United States, more than 6 million adults live with HF, and contemporary estimates suggest that the residual lifetime risk of developing HF is approximately 1 in 4 [5]. HF also contributes to cardiovascular mortality, with over 45% death events from CVD are attributed to HF. Notably, mortality from HF varies by population and phenotype, underscoring the need to better understand age-related risk factors and comorbidities that shape disease development and progression [5]. The prevalence of HF in the aging population in the United States is 1.9% to 2.6% and is expected to further increase to 8.5% of individuals aged 65 to 70 [6]. Moreover, the risk of developing HF among the elderly population (≥60 years of age) is 20 times higher compared to population under 60 years of age [6]. The etiology of HF is variable and extensive.

HF is influenced by both risk factors that contribute to disease development and comorbidities that frequently coexist with HF and worsen disease progression and clinical outcomes [7,8,9,10,11,12,13,14,15,16,17,18,19,20,21,22,23,24,25,26,27]. Although risk factors and comorbidities can be distinguished, some conditions may function as both risk factors and comorbidities (Table 1).

Coronary artery disease (CAD), the primary underlying cause of ischemic heart disease (IHD), is the most common risk factor of HF [28]. Additionally, myocardial infarction (MI) and cardiomyopathy are major risk factors and leading causes of HF, as both can directly impair cardiac structure and function through ischemic injury and ventricular remodeling [11,12]. Other important causes and risk factors for HF include hypertension, diabetes mellitus, obesity, and smoking, all of which can contribute to structural and functional cardiac dysfunction leading to HF development [11,12]. These risk factors share pathological mechanisms, including inflammation, oxidative stress, atherosclerosis, and metabolic dysfunction, which promote structural changes leading to HF development [12]. In addition to traditional risk factors, aging-related conditions such as chronic kidney disease, frailty, cognitive impairment, depression, and structural changes contribute to cardiovascular disease risk in older adults [29]. These findings show that both traditional and non-traditional cardiovascular risk factors contribute to HF development and highlight the importance of prevention strategies, lifestyle modification and risk factor control across the life course [29,30].

Common HF comorbidities include hypertension, atrial fibrillation (AF), chronic kidney disease (CKD), diabetes mellitus, chronic obstructive pulmonary disease (COPD), obesity, ischemic heart disease, and hyperlipidemia [8,26]. Among these, CKD and COPD are recognized as major comorbidities that frequently coexist with and contribute to the progression of HF [16,31]. Older HF patients commonly present with multiple coexisting conditions, including hypertension, AF, diabetes mellitus, COPD, and CKD [32,33]. Additionally, multimorbidity is considered a key feature of HF, regardless of subtype, with many older patients presenting with five or more chronic conditions [33]. This is clinically important because multimorbidity in HF is strongly associated with increased hospital readmission risk, HF-related hospitalization, all-cause mortality, and overall poorer clinical outcomes [26,32,33,34,35]. HF with preserved ejection fraction (HFpEF) is often characterized by a greater burden of non-cardiac comorbidities compared with HF with reduced ejection fraction (HFrEF) and is associated with more non-HF-related hospitalizations and worse clinical outcomes [36].

While there is currently no cure for HF, other than heart transplant, prevention, early diagnosis and appropriate treatment strategies can help improve both life expectancy and quality of life for the elderly [37,38,39]. Additionally, elderly patients often experience various barriers to quality health care. Elderly patients often face various struggles in pharmacological treatments and lifestyle changes. Some examples include medication instructions being too complex and in small print, facing struggles with managing timing and dosage medications and renewing medication supplies [40]. Low income, restricted access to health care and inadequate social support can make it difficult for the elderly to adopt necessary lifestyle changes [41]. In this review, we will provide an overview of the key risk factors associated with HF in the aging population and will explore emerging research on the prevention and early detection of HF, with a focus on managing these risk factors in the elderly population.

## 2. Cardiac Aging

**Figure 1:**
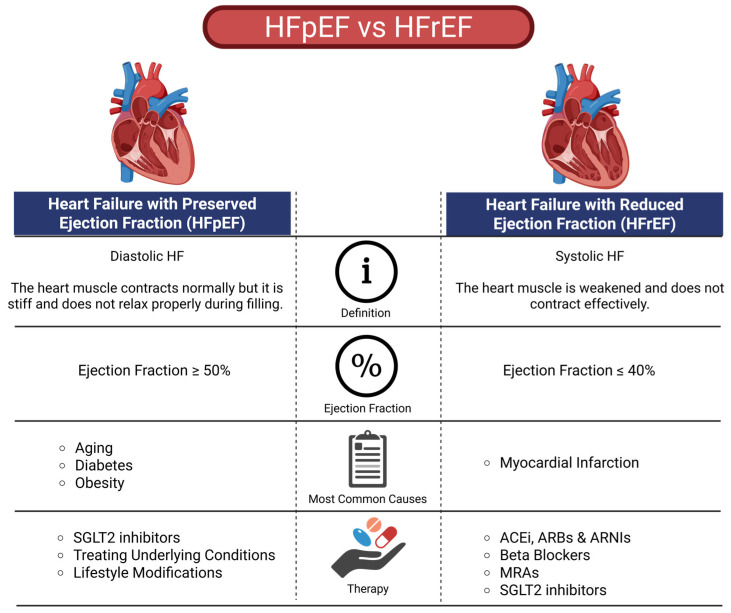
Schematic representation of HFpEF Vs. HFrEF. Created with BioRender.com. Retrieved from https://app.biorender.com/citation/6a17bedbf51650827e93db76 (accessed on 28 May 2026).

Cardiac aging is a key contributor associated with HF development in elderly population [9]. As individuals age, the natural changes in heart structure and function increase the risk of developing HF. The aging process contributes to a decline in the ability of the heart to pump blood efficiently, a condition exacerbated by factors like high blood pressure, coronary artery disease, and other chronic health conditions common in older adults [42,43]. Additionally, age-related changes, such as the stiffening of the heart muscle and blood vessels, as well as a decrease in the responsiveness of the heart to stress, further elevate the likelihood of HF in older individuals [44,45,46]. Figure 1. Patients with symptoms of HF are classified according to left ventricular ejection fraction (LVEF). Heart failure with preserved ejection fraction (HFpEF) is defined by preserved LVEF (≥50%) and heart failure with reduced ejection fraction (HFrEF) is defined by reduced LVEF (≤40%) [4]. Aging associated with both HFpEF and HFrEF [47,48,49].

The average prevalence of HF in individuals aged 70–74, was 1.5% [50]. However, the prevalence increased with age to 5.2% in those aged 80–84 and further increased to 7.2% in those aged 85 and older [50]. Same trend was observed in a study that analyzed participants from the Framingham Heart Study (FHS), Prevention of Renal and Vascular End-stage Disease Study (PREVEND), and Multi-Ethnic Study of Atherosclerosis (MESA) cohorts. Participants were categorized into four age groups: young (<55 years), middle-aged (55–64 years), old (65–74 years), and elderly (≥75 years). In this study, HF incidence was observed in 1% of all young participants, 5% in middle-aged participants, 10% in old participants and 18% in elderly participants [30]. The PREDICTOR study conducted in Central Italy explored the prevalence of HF and left ventricular dysfunction (LVD) [51]. The study found that the overall prevalence of HF was 6.7%, and LVD was common in 46% of the population diagnosed with HF. When segregated by sex, systolic LVD was more common in men (5.1%) than women (1.4%), and diastolic LVD affected 46.1% of participants and increased with age, with no significant gender differences [51].

Multiple studies have been performed to record the mortality rates of HF in the elderly population. One study that observed mortality related to HF in older adults (aged 75 and above) from 1999 to 2019 found that over the 20-year period of the study, there were 5,014,919 deaths recorded due to HF. The age-adjusted mortality rate (AAMR) for HF-related deaths decreased from 141.0 in 1999 to 121.3 in 2019 with an annual decline of 2.1% from 1999 to 2012. However, between 2012 and 2019, the AAMR began to rise again, increasing by 1.7% annually [52]. This observation can be explained by the increasing incidence of comorbidities associated with HF in the aging population, including obesity and diabetes [52].

Interestingly, different types of HF exhibit different rates of mortality. When examining survival rates of patients with isolated systolic HF (when the heart does not contract properly), survival rates over a 10-year period were demonstrated at 39%. However, in the case of isolated diastolic HF (when the heart does not relax properly), the prognosis is relatively better with survival rates of 57% [53]. Notably, elderly individuals can be diagnosed with HF at different stages of the disease. In one study that aimed to classify elderly patients into different stages of HF according to their clinical characteristics, individuals were divided into stage A (at risk for HF but without structural heart disease or symptoms), stage B (structural heart disease without symptoms), and stage C (structural heart disease with symptoms of HF). Individuals with advanced HF stages (B and C) were found to be older and with worse overall health. Plasma markers such as N-terminal pro-B-type natriuretic peptide (NT-proBNP), troponin-I, and (Mid-Regional Pro-Adrenomedullin) MR-proADM were elevated in advanced HF stages and quality of life was worse in individuals in stage C, as reflected by a higher Minnesota Living with Heart Failure Questionnaire (MLHFQ) score. Additionally, individuals at stage C had a higher risk of hospitalization due to symptomatic HF [54]. Survival rates between stages of HF also declined progressively, with Stage 0 showing a 5-year survival rate of 98.9%, which dropped to 74.6% in Stage C. Mortality risks increased across HF stages, with a 1.7-fold increase from stage A to B and a 9.6-fold increase from stage B to C. Men had higher mortality risks than women, especially in stages B and C, with 4–6 times greater risk [55].

Interestingly, while NT-proBNP remains a critical marker for HF, new proteins are emerging as predictors of HF and were explored in the elderly population. In a study examining data from two cohorts: Prospective Investigation of the Vasculature in Uppsala Seniors (PIVUS) and Uppsala Longitudinal Study of Adult Men (ULSAM), additional potential circulating proteins were examined as predictors of HF in ageing individuals. Worse left ventricular systolic function was linked to proteins, such as growth differentiation factor 15 (GDF-15), urokinase-type plasminogen activator surface receptor (U-PAR), matrix metalloproteinase-12 (MMP-12), tumour necrosis factor-related apoptosis-inducing ligand receptor 2 (TRAIL-R2), and spondin-1 (SPON1). However, T-cell immunoglobulin and mucin domain 1 (TIM-1) was the only protein associated with worsened diastolic function. Adding these proteins into risk models may help diagnose individuals that are at high risk for HF at an earlier stage, which will improve treatment and survival rates [56]. Cardiac aging is a significant risk factor for HF, as seen by a common trend across various studies that reveals that the incidence and prevalence of HF increase as you age. These findings highlight the need to improve the diagnosis of individuals who are at high risk for HF, such as the elderly population. For instance, new proteins as predictors of HF were explored in the elderly population as an emerging predictor to improve treatments and survival rates among the elderly population.

## 3. Coronary Artery Disease

Coronary artery disease (CAD) is a major risk factor for HF, CAD and subclinical coronary atherosclerosis are strongly associated with the development of incident HF, including both HFpEF and HFrEF [10,57,58,59]. CAD is also an important comorbidity in HF, where it commonly coexists with other conditions and contributes to worse prognosis and clinical complexity [8,23]. In the Multi-Ethnic Study of Atherosclerosis (MESA), progression of coronary artery calcium was independently associated with incident HF over a median follow-up of 9.6 years, with each 10-unit/year increase in calcium progression associated with a 3% higher HF risk [58]. In a related MESA analysis, coronary artery calcium scores greater than 300 were associated with a higher risk of incident HFpEF [59]. CAD is common in patients with HF and is associated with worse long-term outcomes across HF subtypes. In patients with HFrEF, CAD was frequently observed and was linked to higher all-cause mortality compared with those without CAD (55% vs. 33%) [60]. Similarly, in the I-Preserve analysis of patients with HFpEF, 36% had a history of CAD, and these patients had a worse baseline clinical profile, including more diabetes (32% vs. 25%), higher NT-proBNP levels (449 vs. 282 pg/mL), lower LVEF (58% vs. 61%), and more NYHA class III/IV symptoms (82% vs. 77%) [61]. Patients with both CAD and angina also had higher risks of all-cause death, cardiovascular death, and sudden death, suggesting that CAD in HFpEF is associated with worse mortality and cardiovascular outcomes [61]. In another study of patients newly diagnosed with HF during coronary angiography, CAD was present in 44%, while 56% had no CAD or only mild non-obstructive CAD [62]. The 10-year mortality rate was 40%, with slightly higher mortality in patients with LVEF 10–35% compared with those with LVEF 36–49% (42% vs. 37%) [62]. Importantly, CAD was more strongly associated with mortality than LVEF, and excess mortality compared with the general population was greater in HF patients with CAD than in those without CAD [62]. CAD testing appears to be underused in patients with new onset HF, despite CAD being an important contributor to HF outcomes. In the GWTG-HF registry (2009–2015), after patients with prior CAD were excluded, only 37% of patients hospitalized with new-onset HF underwent CAD testing [63]. Among those tested, 54.1% received invasive testing, while 7.3% underwent revascularization, including PCI in 70 patients (64.8%), CABG in 25 patients (23.1%), and both PCI and CABG in 13 patients (12.0%) [63]. Testing was most common in HFrEF (54%), followed by borderline EF (42%) and HFpEF (28%), suggesting that CAD evaluation was particularly limited in HFpEF [63]. Similarly, a retrospective cohort study within Kaiser Permanente Southern California (KPSC) found that only 54.5% of patients hospitalized with new-onset HFrEF underwent CAD testing during hospitalization or within 90 days [64]. Importantly, CAD testing was associated with fewer HF readmissions or deaths over 1.8 years compared with no testing (17.28 vs. 28.24 events per 100 person-years) [64]. Consistent with these findings, another cohort reported that only 34.8% of patients underwent CAD testing within 90 days before or after their first HF diagnosis [65]. Testing was more common in younger patients, men, those with emergency department visits or hospitalization near diagnosis, systolic dysfunction, and cardiovascular risk factors such as hyperlipidemia, obesity, and smoking [65]. In contrast, patients aged ≥ 80 years, women, and Black patients were less likely to undergo CAD testing [65].

CAD is a major contributor to HF progression and adverse cardiovascular outcomes. Treating CAD in patients with HF is an important part of reducing risk and improving prognosis. In the COMPASS trial, patients with chronic CAD or PAD were included, 22% of the participants had a history of HF [66]. Patients with HF had higher rates of cardiovascular death, myocardial infarction, stroke, and overall mortality compared with those without HF [66]. Treatment with rivaroxaban 2.5 mg twice daily plus aspirin 100 mg once daily reduced major adverse cardiovascular events by 32% in patients with HF, compared with 21% in patients without HF [66]. Among HF patients, this combination also reduced all-cause mortality by 34% and stroke by 52% [66]. CABG is another important treatment approach for patients with ischemic cardiomyopathy and reduced ejection fraction. In the STICH trial, adding CABG to medical therapy reduced major causes of death in HF, including a significant reduction in sudden death among patients [67]. Both optimized medical therapy and revascularization strategies may improve outcomes in selected patients with CAD and HF.

## 4. Cardiomyopathy

Cardiomyopathy has been shown to be a risk major risk factor and comorbidity in HF [11,24]. Cardiomyopathies are an important structural cause of HF in older adults because they directly alter left ventricular size, geometry, and contractile function [68,69]. The major forms of cardiomyopathy include dilated, hypertrophic, and restrictive types, each defined by distinct structural and functional changes in the myocardium [69,70]. Additionally, Cardiac aging promotes structural and functional changes in the myocardium that increase susceptibility to cardiomyopathy and HF [71].

Dilated cardiomyopathy (DCM) is defined by left ventricular or biventricular dilatation with systolic dysfunction that is not fully explained by coronary artery disease or abnormal loading conditions [68]. In a large UK electronic health record cohort of approximately 9 million adults, DCM was the most frequently recorded cardiomyopathy. More than half of patients (56%) already had HF at the time of first DCM diagnosis. Additionally, patients without HF at the time of DCM diagnosis showed the highest HF incidence rates of all cardiomyopathy subtypes [72]. In a registry of 953 patients aged ≥ 60 years with non-ischemic DCM, an AGEF score, based on age, glomerular filtration rate, and ejection fraction, predicted both in-hospital mortality and subsequent adverse events, underscoring that advanced age, renal dysfunction, and reduced EF are key determinants of outcome and rehospitalization in elderly patients [73]. In cohorts with very low LVEF (≤19%), non-ischemic DCM patients often remain free from transplant for several years on optimized medical therapy. However, repeated HF hospitalizations are common and contribute significantly to morbidity [74]. Consistent with these observations, transplant studies identify non-ischemic DCM as one of the leading primary indications for adult heart transplantation, highlighting its central role as a cause of end-stage HFrEF in older patients [75].

Hypertrophic cardiomyopathy (HCM) is the most common inherited cardiomyopathy and is not rare in the general population [76]. HCM is characterized by otherwise unexplained left ventricular hypertrophy with a nondilated cavity and usually preserved or supranormal ejection fraction [76,77]. Additional symptoms include exertional dyspnea and congestion, as impaired relaxation and elevated filling pressures can cause symptomatic patients to present with preserved EF, mimicking an HFpEF phenotype [76,77]. Population-based echocardiographic screening in middle-aged and older adults has detected previously unrecognized HCM in roughly 1 in 500 individuals [76]. In a cohort examining the impact of HCM, patients with HCM were older, with a mean age of 72.3 ± 9.7 years, and had larger left atria, which suggests greater atrial remodeling. They also had a higher prevalence of prior HF, which supports the link between HCM-related structural changes and HF burden in older adults [78]. Long-term cohort data show that many patients with HCM survive into old age with relatively low rates of HF death and hospitalization. In one study, 23% survived to at least 75 years of age, up to 96 years, and overall mortality was similar to that of an age-matched general population, with relatively few HF-related deaths [77]. In a multicenter cohort of older HCM patients, with a mean age of 70 years, HCM-related mortality and HF admissions remained modest under contemporary management, and most deaths occurred from non-cardiac causes [79]. Elderly HCM patients, with a mean age of 80 ± 5 years, still had substantial event rates; about half experienced an endpoint of death or major cardiac events over 5 years [80]. Across these cohorts, approximately 3–7% of HCM patients progress to an “end-stage” phase with systolic dysfunction and advanced HF that may demand transplant evaluation [80].

Restrictive and amyloid cardiomyopathies are less common but highly relevant in the very old, particularly those with transthyretin cardiac amyloidosis (ATTRwt). In a clinical study of older patients, with a mean age of 75 years, with increased left ventricular wall thickness and HF, ATTRwt was frequently identified and typically presented with preserved EF, concentric thickening, restrictive filling, and HF symptoms consistent with an HFpEF phenotype [81]. A multicenter European study of elderly HF patients, with a median age of 82 years, found transthyretin amyloid cardiomyopathy in approximately 13–19% of individuals aged ≥ 80 years with increased wall thickness or HFpEF, indicating that ATTRwt is a major cause of HF in this age group [82]. Autopsy studies further demonstrate the high background prevalence of cardiac amyloid in elderly patients. In a population-based autopsy cohort of individuals aged ≥ 85 years, wild-type transthyretin amyloid deposits were present in about one quarter of hearts [83]. A similar observation was reported in a post-mortem study of unselected patients aged ≥ 75 years, with a median age of 86 years, which found cardiac amyloidosis in 43% of hearts [84]. Clinical and trial data highlight the heavy HF-hospitalization burden associated with transthyretin cardiomyopathy. In a large natural-history cohort of ATTRwt, with a median age of 75 years, HF was present in two-thirds of patients. Median survival from diagnosis was only about 3.6 years, with many deaths preceded by repeated HF admissions [85].

Taken together, these data show that cardiomyopathy subtypes have distinct relationships with HF and hospital burden in older adults. DCM most often produces HFrEF and is strongly associated with incident HF, recurrent HF hospitalizations and the need for heart transplantation in elderly patients [72,73,74]. HCM is relatively common but usually compatible with survival into advanced age, with lower rates of HF hospitalization and only a small minority progressing to end-stage HF [76,77,79,80]. In contrast, transthyretin amyloid cardiomyopathy and other restrictive forms are concentrated in very old adults and are associated with high rates of HF admission and mortality, often presenting with HFpEF-like physiology [81,82,83,84,85,86].

Recognizing the underlying cardiomyopathy subtype in an elderly patient with HF is important for accurate HF phenotyping and for selecting therapies that can reduce HF hospitalizations and improve survival. Studies focusing on older adults show that DCM remains a major driver of HF events and admissions. In a Japanese study of patients diagnosed with DCM at age ≥ 65 years, with an age range of 65–83 years, 5- and 10-year event-free survival from cardiac death or transplant improved markedly after the introduction of ACE inhibitors and β-blockers, indicating better long-term outcomes despite ongoing HF events [87]. Mavacamten is a selective cardiac myosin inhibitor developed to reduce excessive contractility and improve symptoms in HCM [88]. In participants with a mean age of 58.5 years, mavacamten improved functional status, with 65% of patients improving by at least one NYHA class compared with 31% on placebo. By the end of treatment, 50% of mavacamten treated patients reached NYHA class I, compared with 21% on placebo, showing improvement in HF-related symptoms [88]. In the ATTR-ACT trial of patients with transthyretin amyloid cardiomyopathy, with a mean age of 74 years, tafamidis reduced both all-cause mortality (29.5% vs. 42.9%) and the rate of cardiovascular-related hospitalizations (0.48 vs. 0.70 per patient-year) compared with placebo over 30 months, demonstrating that HF hospitalizations are frequent in this population and that disease-modifying therapy can meaningfully reduce them [86].

## 5. Myocardial Infarction (MI)

Another risk factor and comorbidity for HF is myocardial infarction [8,12]. Adverse cardiac remodeling post—MI significantly increases the development of HF in the elderly. Ischemia following an MI event result in cardiomyocyte death, this loss is often irreversible. While reperfusion restores blood flow, it contributes to further injury through the generation of reaction oxygen species. Furthermore, the inflammatory response to myocyte death exacerbates myocardial damage and remodeling [89,90,91]. As for HF subtype, myocardial infarction is more strongly associated with HFrEF [92]. An increased risk for HF following MI in elderly patients was linked to a greater tendency for left ventricular (LV) remodeling. A study that monitored 266 patients with anterior MI over time found that LV remodeling, indicated by an increase in LV end-diastolic volume after 1 year, was observed across all age groups but did not differ significantly with age. However, heart failure hospitalization rates were higher in older patients, with rates of 1.9%, 1.5%, 11.0%, and 20.3% for patients under 48, 48–57, 58–71, and over 71 years, respectively, underscoring age as a significant predictor of HF re-hospitalization [93]. With improvement in early diagnosis, a reduction in- hospital mortality from MI is more common, but the incidence of in-hospital HF has increased [94]. In-hospital HF is more common in older patients and is also correlated with longer hospital stays [95]. A study that analyzed the burden and timing of HF post MI found a direct correlation between HF occurrence and age, with incidence rates of 8.9%, 15.2%, and 25.6% among young, middle-aged, and elderly men, respectively. A similar trend was observed in women but with higher incidence rates than in men, at 10.2%, 16.8%, and 27.1%, in same age groups respectively [95]. Similar trend is seen in patients hospitalized with non-ST-segment elevated myocardial infarction (NSTEMI) and ST-segment elevation myocardial infarction (STEMI). Among NSTEMI patients, 4% (4348 out of 110,128) developed HF, while 3.6% develop HF in STEMI (2813 out of 77,675). Interestingly, however, patients who developed HF were older in both STEMI and NSTEMI compared to patients who did not develop HF. HF development in both groups was associated with worse outcomes, including higher in-hospital mortality, longer hospital stays, and lower rates of optimal discharge interventions [96]. The incidence of new HF has also been observed post-MI in elderly patients, with 75.9% developing HF during the five-year follow-up [94].

A significant proportion of MI cases, especially among the elderly, are asymptomatic or clinically unrecognized MI. This affects 21–33% of men and 26–54% of women [97]. Unrecognized MI carries a risk of cardiovascular death and total mortality that is equal to or greater than that of recognized MI. However, there has been limited research on the cardiovascular morbidity that follows unrecognized MI, including HF. One study looking at unrecognized MI and the development of HF found that in men, HF incidence was higher in recognized MI (39%) than unrecognized MI (37%). Similar trend was found in women, with incidence of HF higher in recognized MI (28%) than unrecognized MI (13%). The risk of HF was significantly increased for both recognized (HR 2.6) and unrecognized (HR 2.4) MI in men. For women, recognized MI was associated with a three-fold higher risk of HF (HR 2.8). However, unrecognized MI in women was not significantly associated with an increased risk of HF with an HR of 1.2 [97].

Risk for HF mortality is increased with age. 70.6% of patients aged 65–69 years developed HF post MI, with 55.7% dying within five years. 75.1% of those aged 70–75 years developed HF post MI, with 61.7% mortality rates in the five years post diagnosis. In patients older than 75 years, 76.8% developed HF with a 70.3% mortality rate [94]. In male patients surviving their first MI, 6% (young), 15% (middle-aged), and 40% (elderly) were later hospitalized or died from heart failure. Female patients had similar rates which also increased with aging. The risk for post-MI HF peaks in the first month’s post MI, declines by 1 year post MI, and remains stable thereafter. This trend is observed similarly between all age groups and sexes [95]. Percutaneous coronary intervention (PCI) is the most common treatment after MI. The success rate of PCI, defined as final thrombolysis in myocardial infarction (TIMI) flow grade of 3. A study evaluating outcomes of elderly patients with acute MI (AMI) complicated by HF among patients who underwent PCI found that higher mortality rates are associated with lower left ventricular ejection fraction (LVEF), longer door-to-balloon times, and final TIMI flow grade less than 3. In contrast, higher LVEF and participation in cardiac rehabilitation were linked to lower mortality rates among elderly patients [98].

Intensive management of risk awareness combined with cardiac rehabilitation programs is important for reducing risk for HF post MI in elderly patients. A study conducted on 101 elderly patients suffering from AMI and comparing medication possession ratio (MPR), cardiac function, self-care abilities, quality of life, adverse events, and patient satisfaction showed that after care intervention, individuals who received intensive risk awareness management and cardiac rehabilitation nursing had improved cardiac function, with better left ventricular measurements and higher ejection fraction, and lower incidence of adverse cardiac events. This observation underscores the importance of intensive risk awareness management combined with cardiac rehabilitation nursing especially in the aging population [99]. These studies demonstrate the many cases in which MI is linked to HF in the elderly population. This shows the importance for interventions such as intensive management of risk awareness combined with cardiac rehabilitation programs.

## 6. Hypertension

Hypertension is another well-established risk factor and comorbidity for HF, particularly in the elderly [8,12,25]. Hypertension induces many functional and morphological changes in the heart due to chronic pressure overload, including cardiac remodeling, myocardial fibrosis, left ventricular hypertrophy (LVH), and both systolic and diastolic dysfunction [100,101,102,103]. Hypertension leads to HFpEF but can progress to HFrEF over time [104]. The link between increased risk for HF in the elderly population and hypertension is best exemplified in a study that looked at the sex specific incidence of HF in elderly hypertensive patients. In this study, men with hypertension were shown to have significantly higher risk for HF (7.86/1000) compared to women with hypertension (4.83 per 1000). Mortality rates in hypertensive patients were also observed to be higher in men (200.9 per 1000 PY) compared with women (113.0 per 1000 PY). Median survival rate after HF diagnosis was 3.94 years, with women having a better prognosis (6.06 vs. 3.32 years) [105]. Prehypertension stage has also been observed to increase the risks of HF. One study that examined elderly patients (mean age 72.8) without prevalent HF and not receiving any anti-hypertensive drugs found that prehypertensive patients (120–139 mm Hg) have similar risk for HF as stage 1 hypertension (140–159 mm Hg), or stage 2 hypertension (≥160 mm Hg). Interestingly, however, increasing systolic blood pressure (SBP) was associated with higher HF risk in women than in men, suggesting a sex specific risk for HF in elderly patients with hypertension [106].

Antihypertensive drugs were associated with better long-term cardiac outcomes with a HR of 0.42 for mortality rate and reduced re-hospitalization rate (HR 0.56). Discharge BP alone was not a strong predictor of 1-year mortality or re-hospitalization among very old patients hospitalized for acute HFpEF. However, extreme BP values were correlated with higher mortality rates with a J-curve pattern [107]. Urapidil, a sympatholytic antihypertensive drug was compared with nitroglycerin in elderly patients with acute decompensated heart failure (ADHF) and hypertension. Both medications significantly reduced systolic and diastolic BP and heart rate over a 7-day treatment period, with significantly decreased NT-proBNP levels from both drugs. However, urapidil also demonstrated greater effectiveness in improving LVEF (55.3%) compared to nitroglycerin (45.2%) [108]. When comparing sacubitril/valsartan (sac/val) normally used for treating HF, with renin-angiotensin-aldosterone system inhibitors (RAASi) in hypertension treatment, sac/val was shown to be more effective in controlling blood pressure, improving heart function, and reducing cardiovascular events compared to RAASi [109]. Hypertension is recognized as a significant risk factor for HF, particularly in the elderly and interventions are key. Nevertheless, additional prospective studies are needed to assess other hypertensive drugs and their effect on HF and mortality rates in the older population.

## 7. Diabetes

Diabetes mellitus is recognized as a risk factor and comorbidity for heart failure [8,12,25,26]. Chronic hyperglycemia further promotes remodeling by activating mast cell and stimulating the release of pro-inflammatory mediators that impair endothelial function. Insulin resistance can contribute to the development of heart failure through mechanisms involving oxidative stress and increase inflammation [110,111]. Diabetes is associated with both subtypes but appears to be a greater susceptibility to HFpEF [112]. In a cohort study focusing on elderly patients with type 2 diabetes, the prevalence of HF was 30.6%, with higher prevalence in women (31%) than men (24.8%) [113]. In another study analyzing hospitalizations due to HF, and classifying by HF subtype, diabetes as a risk factor was present in 44% of HF cases, with slightly higher rates in heart failure with mid-range ejection fraction (HFmrEF) (46.7%) and heart failure with preserved ejection fraction (HFpEF) (45.5%) subtypes, compared to heart failure with reduced ejection fraction (HFrEF) (41.8%). Diabetic HF patients were generally “younger” (72 yrs old) compared to non-diabetic HF patients (77 years old) [114]. From 2005 to 2015, the prevalence of diabetes among HF hospitalizations rose from 43.2% to 45.8% and across all HF types, without significant variation by age. In individuals with newly diagnosed HF, diabetes was more prevalent in HFpEF and HFbEF patients (39.9% each) than in HFrEF (33.1%) [114].

Multiple studies have shown that diabetes contributes to adverse cardiac remodeling which increases the risk of HF in the elderly patients with diabetes. The Northern Manhattan Study (NOMAS) demonstrated that patients with diabetes have significantly higher Left Ventricular (LV) mass, wall thickness and LV diastolic dimension compared to control groups, which may be a contributor to HF [115]. Another parameter linked to the risk of HF in diabetic patients is the expansion of the myocardial extracellular matrix, as measured by the extracellular volume fraction (ECV). A study that observes the ECV in diabetic patients found that ECV was significantly higher in the diabetic population compared to controls with values of 30.2% and 28.1%, respectively. Additionally, elevated ECV was strongly associated with an increased incidence of HF and higher mortality rates in diabetic patients [116]. A population study investigating the effects of type 2 diabetes on the concentric remodeling of the left ventricle revealed significant alterations in cardiac structure and function [117]. Patients with type 2 diabetes exhibited 16% lower left ventricular end diastolic volume compared to controls. This results in a higher ratio of LV mass to LV end—diastolic volume which suggests concentric LV remodeling in individuals with diabetes. Additionally, myocardial triglyceride levels were found to be two-fold higher in the diabetic group compared to controls. Myocardial triglycerides accumulation was also positively correlated with concentric remodeling, suggesting that cardiac steatosis may contribute to the development of HF through concentric LV remodeling and impaired contractile function [117]. Echocardiography has been an important diagnostic tool for HF. One study emphasized the role of echocardiographic measurements, such as global longitudinal strain (GLS), left ventricular hypertrophy (LVH), and left atrial enlargement (LAE), in predicting HF and mortality rates in elderly asymptomatic patients with type 2 diabetes. Impaired GLS was identified to have a better predictive ability than other traditional clinical and echocardiographic markers when predicting incident of HF. The cumulative incidence of heart failure was higher in patients with GLS less than 16%, highlighting the importance of GLS in predicting HF [118].

Glycemic control in diabetic patients has been shown as a crucial strategy in reducing the risk of HF in diabetic patients. A nationwide study conducted in Sweden examining the risk of HF in individuals with type 1 diabetes found that these individuals were four times more likely to be hospitalized with HF compared to age- and sex-matched controls. The risk of developing HF was significantly higher in individuals with poor glycemic control [119]. Further, blood glucose markers like Hemoglobin A1C (HbA1c) have been linked to HF. The Action to Control Cardiovascular Risk in Diabetes (ACCORD) trial which analyzed HbA1c levels in patients with type 2 diabetes found that even 1% increase in baseline HbA1c was correlated with 20% increased risk of HF for each level change [120]. Notably, the link between diabetes and HF is not implied on pre-diabetic cases, as pre-diabetes is not an independent risk factor for HF [121]. Nevertheless, these studies suggest that maintaining glycemic control is crucial for reducing HF risk in individuals with type 2 diabetes [120].

Notably, the link between treatment of diabetes and risk for HF was also observed. In this study, Dapagliflozin, a sodium-glucose cotransporter-2 (SGLT2) inhibitor, was found to reduce the risk of cardiovascular death or hospitalization for HF by 17%, with greatest reduction seen in patients with HFrEF [122]. However, whether the beneficial effect of Dapagliflozin on reducing HFrEF is age dependent and may benefit the elderly population is yet to be discovered. Across studies examining the prevalence of HF among those with diabetes, it is revealed that there is a higher prevalence of HF among those with diabetes. Furthermore, multiple studies have demonstrated that diabetes contributes to adverse cardiac remodeling, which increases the risk of HF, particularly in diabetic elderly patients. The significant role of diabetes in HF emphasizes the need for effective interventions. Various strategies, such as echocardiography and glycemic control, to diagnose and reduce the risk of HF in diabetic patients are emerging interventions to improve treatment outcomes.

## 8. Obesity

Obesity is another major risk factor for HF in the aging population [10,25,123]. Obesity contributes to heart failure through multiple mechanisms. Excess adipose tissue increases systemic inflammation, which can affect the myocardium through pro-inflammatory signaling. This chronic inflammatory state promotes endothelial dysfunction, vasoconstriction, and elevated blood pressure. In addition, increased aldosterone levels activate cardiac mineralocorticoid receptors driving fibrosis and hypertrophy [124,125,126]. Obesity is strongly linked to both phenotypes but demonstrates a stronger association with HFpEF [127]. In a study with elderly cohort, BMI was found to be a strong predictor of LV mass and diastolic dysfunction [128]. Elderly participants with higher BMI have worse diastolic function, characterized by lower E/A ratios and higher E/E’ ratios. Sex-specific differences have also been observed with men having significantly lower E/A ratio and women showing a higher E/E’ ratio. Obesity was found to significantly increase the risk of diastolic dysfunction, with each unit increase in BMI raising the odds by 4% [128]. Obesity is associated with a 138% increase in hospitalization episodes in elderly patients (45–64 years old). For every 1 kg/m^2^ increase in BMI, there is a 6% rise in the risk of HF, emphasizing the cardiovascular risks of higher BMI levels [129]. Exercise capacity, as measured by peak VO_2_, also plays a role in predicting mortality in HF patients. One study found that patients (mean age 62 ± 12 years) with a predicted peak VO_2_ below 50% had significantly worse survival rates compared to those with higher VO_2_ levels. Additionally, when patients were grouped by peak VO_2_, BMI no longer had a significant impact on survival, suggesting that exercise capacity may be a more important predictor of survival in systolic HF than BMI alone [130]. Waist circumference (WC) can also be used as an alternative predictor of mortality in obese elderly patients [131]. Among participants without chronic heart failure (CHF), a greater WC was associated with increased mortality. In participants with CHF, a higher WC was linked to a greater risk of mortality. For every 1 cm increase in WC, the mortality risk increased by 2% in non-CHF individuals and 5% in CHF individuals [131].

Weight management through diet and physical activity has a protective effect on HF risk in the elderly population. Proper diet and exercise contribute to improved cardiac outcomes, with diet having a particularly strong impact on fat reduction, muscle quality, and HF specific quality of life (QOL) in the elderly population. However, the combination of diet and exercise had additive effects on exercise capacity, especially for walking distance [132]. In one study, male participants (mean age of 53 years) engaging in vigorous physical activity experienced a 26% lower overall risk of HF, and those who exercised 5–7 times per week had a 36% lower risk compared to inactive individuals. However, while vigorous physical activity reduced both mortality and HF risk, BMI and physical activity did not show significant interaction in predicting HF risk. The highest risk was observed in obese, inactive participants, who had a 293% higher risk of HF compared to lean, active individuals [123]. Another study looked at the effects of resistance training + caloric restriction + aerobic training (RT + CR + AT) or caloric restriction + aerobic training (CR + AT) in elderly patients (age > 60 years) with HFpEF and obesity. Both groups showed reductions in left ventricular mass and arterial stiffness, but no significant differences were observed in epicardial, pericardial, or paracardial fat. Both interventions improved exercise capacity, weight loss, and cardiovascular function, and the addition of resistance training provided further benefits in muscle strength and quality for HFpEF patients with obesity [133]. In a study that looked at body fat composition in elderly obese patients with HFpEF, intra-abdominal fat was considered to be the strongest predictor of both peak VO_2_ and 6MWD, while lower epicardial fat is associated with better exercise tolerance and physical function. In contrast, higher abdominal and thigh fat were linked to impaired physical performance. Abnormal fat distribution, especially intra-abdominal and intramuscular fat, plays a role in exercise intolerance and physical function in HFpEF patients [134]. Surgical weight loss interventions also show benefits in reducing HF risk. A study comparing gastric bypass surgery to lifestyle modification programs revealed that the surgery group experienced significantly greater weight loss and a reduction in HF risk compared to the lifestyle group. Additionally, a 10 kg weight loss was associated with a reduction in HF risk, further emphasizing the role of weight management in preventing heart failure [135]. These studies highlight the importance of preventive measures to manage obesity, ultimately reducing the risk for HF.

## 9. Arrhythmia

Arrhythmia is recognized as a risk factor and comorbidity for HF [8,10,13,26]. Arrhythmia is an abnormal heart rhythm caused by disruption of the heart’s normal electrical conduction pathway. Atrial fibrillation (AF) and HF are closely linked through a reciprocal relationship [13]. Both conditions arise from and contribute to common pathological processes [136,137]. HF promotes AF through elevated atrial pressure, myocardial fibrosis, and electrical remodeling [138]. Arrhythmia, particularly AF, is associated with both HFpEF and HFrEF, but is more commonly observed in HFpEF [136,138]. In contrast, AF further impairs cardiac function by reducing ventricular filling, worsening fibrosis, and activating the renin–angiotensin–aldosterone system [138]. This overlap means the two conditions often occur together, creating a cycle that leads to poorer outcomes. In a longitudinal cohort (mean age 58 years), AF and HF showed a strong bidirectional relationship. AF is more common in HFpEF than HFrEF, suggesting a stronger association with the HFpEF phenotype [139]. HF increases the risk of developing AF by ~10-fold, while AF elevates the risk of both HF subtypes and is linked to higher mortality, highlighting the reciprocal relationship between these diseases [139]. The use of circulating biomarkers provides an effective approach for predicting risk in AF and HF. In the COMBINE-AF study (median age 71 years), patients with persistent or permanent AF had higher biomarker levels than those with paroxysmal AF [138]. NT-proBNP, hs-cTnT, and GDF-15 were independently associated with increased risk of cardiovascular death or HF hospitalization. Additionally, adding hs-cTnT or GDF-15 to NT-proBNP improved risk prediction, where higher levels meant greater risk and lower levels indicated lower risk even when NT-proBNP was high [138]. In the HEARTS registry (mean age 61.3 years), patients with chronic HF were evaluated [140]. In-hospital ventricular arrhythmia (VA) occurred in 4.2% of HF patients. VA patients have worse hemodynamics and more severe cardiac disease, including greater left ventricular dysfunction and coronary artery disease. Importantly, VA was linked to poorer outcomes, with higher complication rates, increased recurrence of HF, and significantly greater short- and long-term mortality compared to non -VA patients [140].

In the CASTLE-AF trial (median age 65 vs. 63 years), catheter ablation was superior to antiarrhythmic drug therapy in maintaining sinus rhythm in AF patients with HF [141]. This study observed a reduction in AF burden, mortality, and HF hospitalizations, with clear benefits evident by 6 months [141]. Similarly, in HFpEF patients with predominantly persistent AF, catheter ablation improved cardiac function and symptoms compared to medical therapy [136]. These patients had improved filling pressures and natriuretic peptides while increasing cardiac output and quality of life [136]. Notably, 50% of ablated patients no longer met HFpEF criteria versus 7% with medical therapy, suggesting partial reversal of the HFpEF phenotype, particularly in persistent AF [136]. In obesity-related HFpEF, AF is common and shows a more severe phenotype with higher NT-proBNP and worse symptoms despite similar BMI and LVEF [142]. Treatment with semaglutide improved outcomes regardless of AF, with greater benefits in AF patients, suggesting they represent a group that may have enhanced therapeutic benefit [142].

## 10. Chronic Kidney Disease

Chronic kidney disease (CKD) is a major cardiovascular risk factor and is highly prevalent in HF, where it is associated with greater comorbidity burden, worse symptoms, and increased mortality [14,15]. CKD patients have higher risk of HF compared to the general population despite similar risk factors (e.g., hypertension, diabetes) and is a strong predictor of adverse HF outcomes as kidney function declines [143,144]. Evidence from recent studies suggests that CKD is strongly associated with HFpEF, although it is also common across other HF subtypes [145,146]. Both kidney dysfunction (estimated glomerular filtration rate; eGFR) and kidney damage (albumin-to-creatinine ratio; UACR) independently contribute to incident HF and adverse cardiac remodeling [147]. In the ARIC (Atherosclerosis Risk in Communities) cohort (mean age 76 ± 5 years), lower eGFR was associated with increased HF risk but attenuated after adjustment, whereas higher UACR remained a stronger, independent predictor of HFrEF and HFpEF with nonlinear relationships. Additionally, lower eGFR and higher UACR were also linked to higher mortality and adverse cardiac remodeling, including worsened diastolic function and structural changes [147]. In a large CKD cohort, 1774 HF hospitalizations occurred (5.8 per 100 person-years), increasing with worsening kidney function and albuminuria (up to 9.7 per 100 person-years with UACR ≥ 300 mg/g and 2.1–2.9× higher at lower eGFR). HFpEF had slightly higher first event rates than HFrEF (11.2 vs. 8.3 per 1000 person-years). The hospitalization burden in patients with CKD was substantial (36.9 days per 100 person-years) and increases up to 2–4× with low eGFR and high UACR [148].

Recent clinical trials highlight the growing role of cardiorenal therapies in reducing HF risk and improving outcomes, particularly in high-risk populations such as those with CKD. In the FLOW trial, ~19% had baseline HF (predominantly HFpEF 47.9%), with similar kidney function in HF patients. Semaglutide reduced HF outcomes and cardiovascular death by ~24–29% [149]. Similarly, mineralocorticoid receptor antagonist with finerenone has demonstrated beneficial cardiorenal benefits across a CKD population. In the FIDELITY analysis, finerenone significantly reduced HF-related outcomes, including first HHF, CV death, first HHF and recurrent HHF. These effects were consistent across eGFR and UACR subgroups. The lowest event rates were observed in patients with eGFR ≥ 60 mL/min/1.73 m^2^ and low UACR. In contrast, the highest risk was seen at extreme eGFR values (< or >90 mL/min/1.73 m^2^) and with UACR ≥ 300 mg/g [150]. In addition, incretin-based therapies, such as tirzepatide, show benefits in patients with CKD. Tirzepatide reduced the risk of CV death or worsening HF. Importantly, benefits were similar in patients with and without CKD, although absolute risk reduction was greater in CKD (3.6 vs. 1.6 events per 100 patient-years). Additionally, tirzepatide improved health status, exercise capacity, symptoms, and reduced inflammation and body weight [151]. Collectively, these findings show that targeting cardiorenal pathways can reduce the burden of heart failure. The greatest benefits are observed in higher-risk populations, such as patients with established HF & CKD.

## 11. Chronic Obstructive Pulmonary Disease

Chronic obstructive pulmonary disease (COPD) is an important comorbidity and independent risk factor that contributes to the development of HF in the elderly [16,26]. Patients with coexisting COPD and HF present with a more severe clinical profile and worse outcomes. Across studies, these patients are typically older and exhibit more advanced disease and greater HF severity (e.g., higher NYHA class) [152,153,154]. COPD contributes to both HFpEF and HFrEF but appears particularly important in HFpEF [155]. COPD patients are associated with an increased risk of the primary composite endpoint, cardiovascular death, and all-cause mortality [156]. This is accompanied by increased healthcare utilization, including higher rates of heart failure and all-cause hospitalizations, readmissions, and emergency department visits [152,154]. Additionally, mortality is elevated in patients with HF compared to those without HF, regardless of COPD status or HF subtype [152,153,154]. This increased clinical burden is accompanied by higher healthcare costs. HF patients nearly double the median costs due to hospitalizations (>87% of total costs) and higher readmission rates [154]. In addition to clinical and economic burden, quality of life is also significantly impaired, with COPD patients experiencing greater declines in KCCQ scores [156]. Additionally, these patients are more prone to deterioration and less improvement over time [156].

Several clinical trials have observed treatment for HF in patients with COPD, that show both benefits and challenges. In the DELIVER and DAPA-HF trials, dapagliflozin consistently reduced worsening heart failure events, cardiovascular death, and mortality, while also improving symptoms (KCCQ) in patients with and without COPD [157,158]. Beta-blockers are recommended for HFrEF but their use in COPD is limited due to safety concerns. COPD patients are less likely to receive or reach target doses despite worse outcomes [152]. However, evidence shows beta blockers are safe, do not increase exacerbation, and are associated with improved composite outcomes, especially in patients with more severe systolic dysfunction [152,159]. The FINEARTS-HF trial showed that finerenone reduced worsening HF events and cardiovascular death and improved quality of life in HFmrEF/HFpEF patients [150]. These benefits were consistent in patients with and without COPD, with similar effects across outcomes and symptom improvement (KCCQ) and a greater improvement in NYHA class in patients with COPD [150].

## 12. Inflammaging

Inflammaging describes the chronic low-grade, systemic inflammation that develops with age due to immune dysregulation, oxidative stress, senescent cells, and long-term metabolic and environmental stressors [17]. This is characterized by higher circulating cytokines such as interleukin (IL)-6, IL-1β, tumor necrosis factor (TNF)-α, and C-reactive protein (CRP), which have been linked to cardiovascular aging and HF, particularly HF with preserved ejection fraction (HFpEF) [17].

Rheumatoid arthritis (RA), a common chronic inflammatory disease in older adults, is associated with a significantly increased risk of HF independent of traditional risk factors, with incidence up to ~1.8-fold higher than in non-RA populations [160]. Across large cohorts, RA patients also exhibit a greater burden of HF and worse outcomes, including increased mortality [160,161]. In the Health ABC cohort, with a mean age of 74 years, higher baseline IL-6 and TNF-α levels independently predicted incident HF over nearly 10 years of follow-up, with stronger associations for HFpEF than for HFrEF [162]. In the NHANES cohort, with a mean age of 50.28 ± 18.03 years, the systemic immune-inflammation index (SII), derived from lymphocyte, neutrophil, and platelet counts, was associated with increased HF risk and prevalence, showing a dose-dependent relationship and correlating with greater comorbidity burden [163]. In emergency departments, among patients presenting with acute dyspnea with a mean age of 75 years, IL-6 levels were higher in those with acute heart failure (AHF) compared to non-AHF patients. Elevated IL-6 is a strong biomarker in AHF, independently predicting worse clinical profiles, higher mortality, and adverse outcomes [164]. Inflammatory pathways remain important therapeutic targets, with IL-1β, IL-1, IL-6, and CCL2/CCR2 inhibition showing potential benefit subgroups, although further targeted studies are needed [165,166,167,168,169]. In the CANTOS trial, which evaluated the IL-1β inhibitor canakinumab, higher baseline inflammation was strongly associated with an increased risk of heart failure hospitalization (HHF). Although canakinumab did not reduce HHF overall, a dose-dependent benefit was observed in patients who received 300 mg of the drug. Greater reductions in HHF and improved cardiovascular outcomes were seen in patients who achieved lower hsCRP levels (<2 mg/L) [166]. Although inflammaging describes the age-related chronic inflammatory state that increases susceptibility to HF, chronic inflammation plays a broader and more direct role in HF pathophysiology independent of aging.

## 13. Chronic Inflammation

Chronic Inflammation is an important contributor to HF [18,19]. Elevated pro-inflammatory cytokines, including TNF-α, IL-1, and IL-6, can promote adverse cardiac remodeling, impair contractile function, contribute to microvascular dysfunction, and affect extracellular matrix regulation within the cardiac muscle [165,170]. This is relevant in HFpEF, where inflammation plays a major role in disease development and prognosis. In the MESA cohort, higher IL-6, CRP, and TNF-α levels were associated with a greater risk of developing HF [165]. When HF was separated by subtype, IL-6 and CRP were specifically associated with HFpEF, but not HFmrEF or HFrEF, suggesting that systemic inflammation may be particularly important in HFpEF development [165]. IL-6 also appears to be closely linked to HFpEF severity and outcomes. Higher IL-6 was more strongly associated with cardiovascular mortality in HFpEF than in HFrEF [171]. When IL-6 is combined with NT-proBNP, it showed a stepwise increase in mortality risk [171]. Similarly, in patients with HFpEF, IL-6 was associated with a more severe clinical and biomarker profile, including higher TNF-α, hsCRP, galectin-3, and NT-proBNP, and remained independently associated with all-cause mortality, cardiovascular death, and HF hospitalization [172]. Other inflammatory and immune-related biomarkers have also been linked to worse NYHA class, diastolic dysfunction, and poor outcomes in HFpEF, with GDF-15 showing the strongest positive association with HF hospitalization or all-cause death [173]. A panel of 87 measurable biomarkers was analyzed in patients with HFpEF, with 32 biomarkers associated with NYHA class and several inflammatory and immune-related biomarkers linked to diastolic dysfunction and poor outcomes [173]. Among these, GDF-15 showed the strongest positive association with HF hospitalization or all-cause death [173]. This is consistent with findings in HFrEF, where higher GDF-15 was associated with greater disease severity, higher NT-proBNP and hs-TnT, poorer exercise capacity, and increased mortality or hospitalization risk [174]. In chronic HF patients with renal dysfunction, a higher systemic immune-inflammation index was also independently associated with all-cause mortality, with high-SII patients showing a 70.3% increased mortality risk during follow-up [175].

Inflammation contributes to cardiac remodeling and heart failure progression; targeted anti-inflammatory approaches have been investigated as potential strategies to improve cardiac function [176]. A study looking at IL-1 blockade with anakinra showed that it may improve LV systolic function in patients with systolic heart failure without increasing myocardial oxygen demand. Anakinra increased LVEF from 30% to 36% after 14 days, while placebo showed no significant change. LVEes also increased from 1.0 to 1.3 mmHg/mL, suggesting improved contractility, while pressure-volume area did not increase, indicating no rise in estimated cardiac oxygen consumption [167]. A case study described cardiac AA amyloidosis in a 77-year-old man with long-standing rheumatoid arthritis. After switching to tocilizumab, an IL-6 receptor blocker, both inflammation and cardiac function improved. LVEF increased from 42.0% to 60.4%, LV mass decreased from 196 g to 101 g, and NT-proBNP decreased from 2947 pg/mL to 606 pg/mL after 10 months, with a further decrease to 325 pg/mL after 2 years. These findings suggest that IL-6 blockade may benefit selected patients with inflammation-driven cardiac AA amyloidosis [168]. In the phase 2b CENTAUR trial, cenicriviroc (CVC), an oral CCR2/CCR5 antagonist, was tested in patients randomized to CVC or placebo. CVC reduced inflammatory markers, including hs-CRP, IL-6, fibrinogen, IL-1β, and sCD14, and increased CCL2 and CCL4, confirming CCR2/CCR5 blockade and suggesting potential anti-inflammatory and antifibrotic effects in NASH-related fibrosis [169]. These findings suggest that targeting inflammatory pathways may improve cardiac function and reduce inflammatory activity, although more studies are needed to determine their role in HF treatment.

## 14. Smoking

Smoking is another major risk factor for developing HF in the aging population [12]. Smoking can damage endothelial lining through reduction of nitric oxide production, increase inflammation and oxidative stress. Smoking can also lead to the development of atherosclerosis as plaque builds up in blood vessel walls. The combination of these stressors can lead to the development of HF [177]. According to a study on the trends in current smoking prevalence from 1998–2018, smoking was highest in the younger aged adults ranging from 55–64 compared to the other cohorts that were older [178]. Current and former smokers are found to have a higher incidence and risk for HF compared to individuals who never smoked [179,180,181]. Current smokers were found to have higher risk of HF hospitalization and higher risk of all-cause death compared to former or never smokers. Additionally, current smokers had higher risk of cardiovascular death [182]. In terms of mortality, former smokers had comparable risks of all-cause mortality to never-smokers [183]. Further, greater cumulative smoking exposure significantly increases the incidence of HF. Individuals with over 25 pack-years of smoking had approximately double the HF risk compared to never smokers [184]. Former smokers were linked to HFpEF and current smoking is associated with HFrEF [184]. Calculated HR among current smokers for HFpEF and HFrEF is 2.28 and 2.16, respectively [185]. Additionally, each 10 pack-year increment increased HF risk, with HRs of 1.16 for HFpEF and 1.09 for HFrEF [185]. However, longer smoking cessation was associated with reduced HF risk. After 30 years of cessation, the risk for both HFpEF and HFrEF was similar to never smokers. Individuals who had quit smoking for over 30 years had approximately 50% lower risk for both HF phenotypes compared to current smokers, suggesting that the cardiac damage occurred by smoking is reversible [185].

## 15. Obstructive Sleep Apnea (OSA)

Another risk factor and comorbidity for HF in the aging population is obstructive sleep apnea (OSA) [20,27]. Obstructive sleep apnea is prevalent across both HF subtypes without clear differentiation [20,186]. One study evaluated the effects of in-hospital positive airway pressure (PAP) treatment for OSA in patients with acute decompensated heart failure (ADHF). The findings demonstrated that patients adhering to PAP therapy for 3–4 h per night showed significant improvements in LVEF and reductions in both left ventricular end-systolic volume and end-diastolic volume. These improvements suggest that PAP usage may help reduce afterload in patients who consistently use the therapy. Additionally, higher LVEF at discharge was correlated with a reduced likelihood of hospital readmission [187]. Another study investigated the impact of sleep-disordered breathing (SDB), specifically central sleep apnea (CSA) and OSA, on post-discharge mortality in patients hospitalized with acute heart failure (AHF) and reduced LVEF (≤45%). Both CSA and OSA were identified as independent risk factors for post-discharge mortality, with mortality rates of 34% for CSA patients and 32% for OSA patients within three years post-discharge. Patients who received PAP therapy had significantly better survival rates compared to those untreated, indicating that PAP treatment may reduce mortality risks associated with SDB [188]. In addition to its role in mortality, OSA has also been linked to an increased risk of hospital readmissions in HF patients. One study with an average age of 72 years, found that those with OSA had a higher LVEF than those without OSA. Importantly, OSA emerged as the strongest independent predictor of all-cause hospital readmissions at both 30 and 90 days, with a particularly strong association with heart failure-specific readmissions at 90 days [189].

## 16. Alcohol Consumption

Excess alcohol consumption is a significant risk factor for HF at any age, but with more prevalent risk at older age [21,22]. Alcohol consumption has been linked to the development of cardiovascular disease by elevating blood pressure, serum cholesterol and triglyceride levels. This combination can contribute to the progression of heart failure [190,191]. Alcohol consumption is associated with incident HF in both HFpEF and HFrEF populations [192]. A study looking at the link between amounts of alcohol consumed and risk for HF revealed that heavier drinkers had an increased risk of non-CAD-related HF. Interestingly, however, moderate drinkers had reduced risk of CAD-related HF [193]. This study was further supported by another study performed on participant with an average age of 73.7. In this study, increasing levels of moderate alcohol consumption were associated with attenuated risk of HF among older people [194]. The type of alcohol was also examined, showing that wine consumption was associated with a lower risk of non-CAD-related HF, which may be attributed to higher levels of antioxidants in wine, compared to other alcohols, known for its cardioprotective properties [193]. In sex specific differences in alcohol-related HF risk, men have lower risk of congestive HF at all levels of alcohol consumption compared to non-drinkers, with the lowest risk seen in men consuming 1 to 7 or 8 to 14 drinks per week. In women, however, the risk reduction for congestive HF was observed in those who consumed 3 to 7 drinks per week. Former drinkers, whether male or female, did not show a significant difference in HF risk compared to non-drinkers [195]. These sex specific differences imply differences in alcohol metabolic pathways between men and women which may explain the different risk for HF in response to amount of alcohol consumed.

## 17. Discussion

This review offers a comprehensive analysis of the various risk factors and comorbidities contributing to HF in the elderly population. HF in older adults continues to pose a significant public health challenge, with far-reaching implications for both individual well-being and societal resources. As the global population ages, the incidence, prevalence, and mortality rates associated with heart failure are increasing, further highlighting the urgency of addressing this issue [196]. This review outlines the impact of each risk factor and comorbidities on the development and progression of HF in the elderly, underscoring their profound effects on health outcomes. By identifying and understanding these risk factors and comorbidities, we can develop tailored and effective interventions aimed at mitigating the specific threats posed by HF in the aging population. Such interventions emphasize the need for pharmacological treatments and lifestyle modifications in managing the risk factors and comorbidities for heart failure. Both pharmacological treatments and lifestyle modifications have been shown to significantly reduce the risk of developing HF and improve overall cardiovascular health [39,132,135,197,198].

Looking toward the future, to mitigate the risk and comorbidities for HF in the aging population, it is essential to focus on improving access to preventive measures, ensuring that older adults have the resources and support needed to actively manage their heart health. A key step for individuals looking to engage with preventative strategies is understanding the risk factors and comorbidities they need to manage. Therefore, it is essential to continue developing innovative diagnostic methods, as described in this review, so that individuals can effectively evaluate how to reduce their risk of HF with a focus on prevention.

Furthermore, a concerted effort should be made to increase the inclusion of older patients in heart health management programs and support systems, to help reduce the barriers they may face. By adopting a more comprehensive, prevention-oriented approach, we can improve the quality of life for elderly individuals, reduce the burden of heart failure, and help mitigate the risks associated with aging and cardiovascular health.

In summary, a multifaceted strategy that integrates early identification, individualized care plans, and a focus on prevention through both medications and lifestyle changes holds the key to reducing the impact of heart failure in the aging population. By prioritizing these efforts, we can improve not only the health outcomes of older adults but also ease the economic burden of heart failure on healthcare systems worldwide.

## Figures and Tables

**Table 1:** Risk Factors and Comorbidities Contributing to Heart Failure.

Factors/Conditions	Risk Factor	Comorbidity	References
Cardiac Aging	✓		[9]
Coronary Artery Disease	✓	✓	[8,10,11,23]
Cardiomyopathy	✓	✓	[11,24]
Myocardial Infarction	✓	✓	[8,12]
Hypertension	✓	✓	[8,12,28]
Diabetes	✓	✓	[8,12,25,26]
Obesity	✓	✓	[10,25]
Arrhythmia	✓	✓	[8,10,13,26]
Chronic Kidney Disease	✓	✓	[8,14,15,26]
Chronic Obstructive Pulmonary Disease	✓	✓	[16,26]
Inflammaging	✓		[17]
Chronic Inflammation	✓		[18,19]
Smoking	✓		[12]
Obstructive Sleep Apnea	✓	✓	[20,27]
Alcohol Consumption	✓		[21,22]

## Data Availability

No new data were created or analyzed in this study. Data sharing is not applicable to this article.

## References

[1] Yazdanyar A., Newman A.B. (2009). The Burden of Cardiovascular Disease in the Elderly: Morbidity, Mortality, and Costs. Clin. Geriatr. Med..

[2] Tian F., Chen L., Qian Z.M., Xia H., Zhang Z., Zhang J., Wang C., Vaughn M.G., Tabet M., Lin H. (2023). Ranking age-specific modifiable risk factors for cardiovascular disease and mortality: Evidence from a population-based longitudinal study. EClinicalMedicine.

[3] Qu C., Liao S., Zhang J., Cao H., Zhang H., Zhang N., Yan L., Cui G., Luo P., Zhang Q. (2024). Burden of cardiovascular disease among elderly: Based on the Global Burden of Disease Study 2019. Eur. Heart J. Qual. Care Clin. Outcomes.

[4] Bozkurt B., Coats A.J.S., Tsutsui H., Abdelhamid C.M., Adamopoulos S., Albert N., Anker S.D., Atherton J., Böhm M., Butler J. (2021). Universal definition and classification of heart failure: A report of the Heart Failure Society of America, Heart Failure Association of the European Society of Cardiology, Japanese Heart Failure Society and Writing Committee of the Universal Definition of Heart Failure: Endorsed by the Canadian Heart Failure Society, Heart Failure Association of India, Cardiac Society of Australia and New Zealand, and Chinese Heart Failure Association. Eur. J. Heart Fail..

[5] Abovich A., Matasic D.S., Cardoso R., Ndumele C.E., Blumenthal R.S., Blankstein R., Gulati M. (2023). The AHA/ACC/HFSA 2022 Heart Failure Guidelines: Changing the Focus to Heart Failure Prevention. Am. J. Prev. Cardiol..

[6] Bozkurt B., Ahmad T., Alexander K.M., Baker W.L., Bosak K., Breathett K., Fonarow G.C., Heidenreich P., Ho J.E., Hsich E. (2023). Heart Failure Epidemiology and Outcomes Statistics: A Report of the Heart Failure Society of America. J. Card. Fail..

[7] Agress S., Sheikh J.S., Ramos A.A.P., Kashyap D., Razmjouei S., Kumar J., Singh M., Lak M.A., Osman A., Haq M.Z.U. (2024). The Interplay of Comorbidities in Chronic Heart Failure: Challenges and Solutions. Curr. Cardiol. Rev..

[8] Khan M.S., Tahhan A.S., Vaduganathan M., Greene S.J., Alrohaibani A., Anker S.D., Vardeny O., Fonarow G.C., Butler J. (2020). Trends in prevalence of comorbidities in heart failure clinical trials. Eur. J. Heart Fail..

[9] Goyal P., Maurer M.S., Roh J. (2024). Aging in Heart Failure: Embracing Biology Over Chronology: JACC Family Series. JACC Heart Fail..

[10] Triposkiadis F., Xanthopoulos A., Butler J. (2019). Cardiovascular Aging and Heart Failure: JACC Review Topic of the Week. J. Am. Coll. Cardiol..

[11] Khatibzadeh S., Farzadfar F., Oliver J., Ezzati M., Moran A. (2013). Worldwide risk factors for heart failure: A systematic review and pooled analysis. Int. J. Cardiol..

[12] Meijers W.C., De Boer R.A. (2019). Common risk factors for heart failure and cancer. Cardiovasc. Res..

[13] Ellison K.E., Stevenson W.G., Sweeney M.O., Epstein L.M., Maisel W.H. (2003). Management of arrhythmias in heart failure. Congest. Heart Fail..

[14] Löfman I., Szummer K., Dahlström U., Jernberg T., Lund L.H. (2017). Associations with and prognostic impact of chronic kidney disease in heart failure with preserved, mid-range, and reduced ejection fraction. Eur. J. Heart Fail..

[15] van de Wouw J., Broekhuizen M., Sorop O., Joles J.A., Verhaar M.C., Duncker D.J., Danser A.H.J., Merkus D. (2019). Chronic Kidney Disease as a Risk Factor for Heart Failure with Preserved Ejection Fraction: A Focus on Microcirculatory Factors and Therapeutic Targets. Front. Physiol..

[16] de Miguel Díez J., Morgan J.C., García R.J. (2013). The association between COPD and heart failure risk: A review. Int. J. Chronic Obstr. Pulm. Dis..

[17] Aranda J.F., Ramírez C.M., Mittelbrunn M. (2025). Inflammageing, a targetable pathway for preventing cardiovascular diseases. Cardiovasc. Res..

[18] Van Linthout S., Tschöpe C. (2017). Inflammation-Cause or Consequence of Heart Failure or Both?. Curr. Heart Fail. Rep..

[19] Amara M., Stoler O., Birati E.Y. (2025). The Role of Inflammation in the Pathophysiology of Heart Failure. Cells.

[20] Javaheri S., Javaheri S. (2022). Obstructive Sleep Apnea in Heart Failure: Current Knowledge and Future Directions. J. Clin. Med..

[21] Djoussé L., Gaziano J.M. (2008). Alcohol consumption and heart failure: A systematic review. Curr. Atheroscler. Rep..

[22] Piano M.R., Marcus G.M., Aycock D.M., Buckman J., Hwang C.L., Larsson S.C., Mukamal K.J., Roerecke M., Hu F.B., Kiechl S. (2025). Alcohol Use and Cardiovascular Disease: A Scientific Statement from the American Heart Association. Circulation.

[23] Deichl A., Wachter R., Edelmann F. (2022). Comorbidities in heart failure with preserved ejection fraction. Herz.

[24] Liang L., Sun J., Chen L., Li Z., Zhang W. (2022). Association between comorbid cardiomyopathy and composite endpoints of patients with congestive heart failure in the intensive care unit: A retrospective cohort study. J. Thorac. Dis..

[25] Bozkurt B., Aguilar D., Deswal A., Dunbar S.B., Francis G.S., Horwich T., Jessup M., Kosiborod M., Pritchett A.M., Ramasubbu K. (2016). Contributory Risk and Management of Comorbidities of Hypertension, Obesity, Diabetes Mellitus, Hyperlipidemia, and Metabolic Syndrome in Chronic Heart Failure: A Scientific Statement from the American Heart Association. Circulation.

[26] Scholten M., Davidge J., Agvall B., Halling A. (2024). Comorbidities in heart failure patients that predict cardiovascular readmissions within 100 days-An observational study. PLoS ONE.

[27] Yeghiazarians Y., Jneid H., Tietjens J.R., Redline S., Brown D.L., El-Sherif N., Mehra R., Bozkurt B., Ndumele C.E., Somers V.K. (2021). Obstructive Sleep Apnea and Cardiovascular Disease: A Scientific Statement from the American Heart Association. Circulation.

[28] Chuy K.L., Velazquez E.J., Lansky A.J., Jamil Y., Ahmad Y. (2023). Current Landscape and Future Directions of Coronary Revascularization in Ischemic Systolic Heart Failure: A Review. J. Soc. Cardiovasc. Angiogr. Interv..

[29] Díez-Villanueva P., Jiménez-Méndez C., Bonanad C., García-Blas S., Pérez-Rivera Á., Allo G., García-Pardo H., Formiga F., Camafort M., Martínez-Sellés M. (2022). Risk Factors and Cardiovascular Disease in the Elderly. Rev. Cardiovasc. Med..

[30] Tromp J., Paniagua S.M.A., Lau E.S., Allen N.B., Blaha M.J., Gansevoort R.T., Hillege H.L., Lee D.E., Levy D., Vasan R.S. (2021). Age dependent associations of risk factors with heart failure: Pooled population based cohort study. BMJ.

[31] Van Deursen V.M., Urso R., Laroche C., Damman K., Dahlström U., Tavazzi L., Maggioni A.P., Voors A.A. (2014). Co-morbidities in patients with heart failure: An analysis of the European Heart Failure Pilot Survey. Eur. J. Heart Fail..

[32] Regan J.A., Kitzman D.W., Leifer E.S., Kraus W.E., Fleg J.L., Forman D.E., Whellan D.J., Wojdyla D., Parikh K., O’Connor C.M. (2019). Impact of Age on Comorbidities and Outcomes in Heart Failure with Reduced Ejection Fraction. JACC Heart Fail.

[33] Saczynski J.S., Go A.S., Magid D.J., Smith D.H., McManus D.D., Allen L., Ogarek J., Goldberg R.J., Gurwitz J.H. (2013). Patterns of comorbidity in older adults with heart failure: The Cardiovascular Research Network PRESERVE study. J. Am. Geriatr. Soc..

[34] Lee K.S., Park D.I., Lee J., Oh O., Kim N., Nam G. (2023). Relationship between comorbidity and health outcomes in patients with heart failure: A systematic review and meta-analysis. BMC Cardiovasc. Disord..

[35] Salmon T., Essa H., Tajik B., Isanejad M., Akpan A., Sankaranarayanan R. (2022). The Impact of Frailty and Comorbidities on Heart Failure Outcomes. Card. Fail. Rev..

[36] Ather S., Chan W., Bozkurt B., Aguilar D., Ramasubbu K., Zachariah A.A., Wehrens X.H.T., Deswal A. (2012). Impact of noncardiac comorbidities on morbidity and mortality in a predominantly male population with heart failure and preserved versus reduced ejection fraction. J. Am. Coll. Cardiol..

[37] Pagnesi M., Metra M., Cohen-Solal A., Edwards C., Adamo M., Tomasoni D., Lam C.S.P., Chioncel O., Diaz R., Filippatos G. (2023). Uptitrating Treatment After Heart Failure Hospitalization Across the Spectrum of Left Ventricular Ejection Fraction. J. Am. Coll. Cardiol..

[38] Yu D.S.F., Li P.W.C., Li S.X., Smith R.D., Yue S.C.S., Yan B.P.Y. (2022). Effectiveness and Cost-effectiveness of an Empowerment-Based Self-care Education Program on Health Outcomes Among Patients with Heart Failure: A Randomized Clinical Trial. JAMA Netw. Open.

[39] Kosiborod M.N., Deanfield J., Pratley R., Borlaug B.A., Butler J., Davies M.J., Emerson S.S., Kahn S.E., Kitzman D.W., Lingvay I. (2024). Semaglutide versus placebo in patients with heart failure and mildly reduced or preserved ejection fraction: A pooled analysis of the SELECT, FLOW, STEP-HFpEF, and STEP-HFpEF DM randomised trials. Lancet.

[40] Horvat M., Eržen I., Vrbnjak D. (2024). Barriers and Facilitators to Medication Adherence among the Vulnerable Elderly: A Focus Group Study. Healthcare.

[41] Theodorakis N., Kollia Z., Christodoulou M., Nella I., Spathara A., Athinaou S., Triantafylli G., Hitas C., Anagnostou D., Kreouzi M. (2025). Barriers to Implementing Effective Healthcare Practices for the Aging Population: Approaches to Identification and Management. Cureus.

[42] Lloyd-Jones D.M., Evans J.C., Levy D. (2005). Hypertension in adults across the age spectrum: Current outcomes and control in the community. JAMA.

[43] Franklin S.S., Larson M.G., Khan S.A., Wong N.D., Leip E.P., Kannel W.B., Levy D. (2001). Does the relation of blood pressure to coronary heart disease risk change with aging? The Framingham Heart Study. Circulation.

[44] Pandey A., Kraus W.E., Brubaker P.H., Kitzman D.W. (2020). Healthy Aging and Cardiovascular Function: Invasive Hemodynamics During Rest and Exercise in 104 Healthy Volunteers. JACC Heart Fail..

[45] Pollack M., Phaneuf S., Dirks A., Leeuwenburgh C. (2002). The role of apoptosis in the normal aging brain, skeletal muscle, and heart. Ann. N. Y. Acad. Sci..

[46] Blacher J., Agnoletti D., Protogerou A.D., Iaria P., Czernichow S., Zhang Y., Safar M.E. (2012). Aortic stiffness; inflammation, denutrition and prognosis in the oldest people. J. Hum. Hypertens..

[47] Gök G., Kılıç S., Sinan Ü.Y., Turkoglu E., Kemal H., Zoghi M. (2020). Epidemiology and clinical characteristics of hospitalized elderly patients for heart failure with reduced, mid-range and preserved ejection fraction. Heart Lung.

[48] Gong F.F., Jelinek M.V., Castro J.M., Coller J.M., McGrady M., Boffa U., Shiel L., Liew D., Wolfe R., Stewart S. (2018). Risk factors for incident heart failure with preserved or reduced ejection fraction, and valvular heart failure, in a community-based cohort. Open Heart.

[49] Brouwers F.P., De Boer R.A., Van Der Harst P., Voors A.A., Gansevoort R.T., Bakker S.J., Hillege H.L., Van Veldhuisen D.J., Van Gilst W.H. (2013). Incidence and epidemiology of new onset heart failure with preserved vs. reduced ejection fraction in a community-based cohort: 11-year follow-up of PREVEND. Eur. Heart J..

[50] Danielsen R., Thorgeirsson G., Einarsson H., Ólafsson Ö., Aspelund T., Harris T.B., Launer L., Gudnason V. (2017). Prevalence of heart failure in the elderly and future projections: The AGES-Reykjavík study. Scand. Cardiovasc. J..

[51] Mureddu G.F., Agabiti N., Rizzello V., Forastiere F., Latini R., Cesaroni G., Masson S., Cacciatore G., Colivicchi F., Uguccioni M. (2012). Prevalence of preclinical and clinical heart failure in the elderly. A population-based study in Central Italy. Eur. J. Heart Fail..

[52] Siddiqi T.J., Minhas A.M.K., Greene S.J., Van Spall H.G.C., Khan S.S., Pandey A., Mentz R.J., Fonarow G.C., Butler J., Khan M.S. (2022). Trends in Heart Failure-Related Mortality Among Older Adults in the United States from 1999–2019. JACC Heart Fail..

[53] Olofsson M., Boman K. (2015). Impact on mortality of systolic and/or diastolic heart failure in the elderly—10 years of follow-up. J. Clin. Gerontol. Geriatr..

[54] Gaborit F.S., Kistorp C., Kümler T., Hassager C., Tønder N., Køber L., Hansen P.M., Kamstrup P.R., Faber J., Iversen K.K. (2019). Prevalence of early stages of heart failure in an elderly risk population: The Copenhagen Heart Failure Risk Study. Open Heart.

[55] Ammar K.A., Jacobsen S.J., Mahoney D.W., Kors J.A., Redfield M.M., Burnett J.C., Rodeheffer R.J. (2007). Prevalence and prognostic significance of heart failure stages: Application of the American College of Cardiology/American Heart Association heart failure staging criteria in the community. Circulation.

[56] Stenemo M., Nowak C., Byberg L., Sundström J., Giedraitis V., Lind L., Ingelsson E., Fall T., Ärnlöv J. (2018). Circulating proteins as predictors of incident heart failure in the elderly. Eur. J. Heart Fail..

[57] John J.E., Claggett B., Skali H., Solomon S.D., Cunningham J.W., Matsushita K., Konety S.H., Kitzman D.W., Mosley T.H., Clark D. (2022). Coronary Artery Disease and Heart Failure with Preserved Ejection Fraction: The ARIC Study. J. Am. Heart Assoc..

[58] Bakhshi H., Ambale-Venkatesh B., Yang X., Ostovaneh M.R., Wu C.O., Budoff M., Bahrami H., Wong N.D., Bluemke D.A., Lima J.A.C. (2017). Progression of Coronary Artery Calcium and Incident Heart Failure: The Multi-Ethnic Study of Atherosclerosis. J. Am. Heart Assoc..

[59] Sharma K., Al Rifai M., Ahmed H.M., Dardari Z., Silverman M.G., Yeboah J., Nasir K., Sklo M., Yancy C., Russell S.D. (2017). Usefulness of Coronary Artery Calcium to Predict Heart Failure with Preserved Ejection Fraction in Men Versus Women (from the Multi-Ethnic Study of Atherosclerosis). Am. J. Cardiol..

[60] Anker N., Olesen K.K.W., Thrane P.G., Gyldenkerne C., Mortensen M.B., Nielsen R.R., Løgstrup B.B., Würtz M., Nielsen J.C., Maeng M. (2025). Coronary Artery Disease Doubles Excess Mortality in Patients with Heart Failure with Reduced Ejection Fraction. J. Am. Heart Assoc..

[61] Badar A.A., Perez-Moreno A.C., Hawkins N.M., Jhund P.S., Brunton A.P.T., Anand I.S., McKelvie R.S., Komajda M., Zile M.R., Carson P.E. (2015). Clinical Characteristics and Outcomes of Patients with Coronary Artery Disease and Angina: Analysis of the Irbesartan in Patients with Heart Failure and Preserved Systolic Function Trial. Circ. Heart Fail..

[62] Nielsen R.R., Pryds K., Olesen K.K.W., Mortensen M.B., Gyldenkerne C., Nielsen J.C., Hindricks G., Dagres N., Maeng M. (2024). Coronary Artery Disease Is A Stronger Predictor of All-Cause Mortality Than Left Ventricular Ejection Fraction Among Patients with Newly Diagnosed Heart Failure: Insights from the WDHR. J. Am. Heart Assoc..

[63] O’Connor K.D., Brophy T., Fonarow G.C., Blankstein R., Swaminathan R.V., Xu H., Matsouaka R.A., Albert N.M., Velazquez E.J., Yancy C.W. (2020). Testing for Coronary Artery Disease in Older Patients with New-Onset Heart Failure: Findings from Get with The Guidelines-Heart Failure. Circ. Heart Fail..

[64] Huang C.W., Kohan S., Liu I.L.A., Lee J.S., Baghdasaryan N.C., Park J.S., Vallejo J.D., Subject C.C., Nguyen H., Lee M.S. (2024). Association Between Coronary Artery Disease Testing in Patients with New-Onset Heart Failure and Heart Failure Readmission and Mortality. J. Gen. Intern. Med..

[65] Zheng J., Heidenreich P.A., Kohsaka S., Fearon W.F., Sandhu A.T. (2022). Variability in Coronary Artery Disease Testing for Patients with New-Onset Heart Failure. J. Am. Coll. Cardiol..

[66] Branch K.R., Probstfield J.L., Eikelboom J.W., Bosch J., Maggioni A.P., Cheng R.K., Bhatt D.L., Avezum A., Fox K.A.A., Connolly S.J. (2019). Rivaroxaban with or Without Aspirin in Patients with Heart Failure and Chronic Coronary or Peripheral Artery Disease. Circulation.

[67] Carson P., Wertheimer J., Miller A., O’Connor C.M., Pina I.L., Selzman C., Sueta C., She L., Greene D., Lee K.L. (2013). The STICH trial (surgical treatment for ischemic heart failure): Mode-of-death results. JACC Heart Fail.

[68] Richardson P., McKenna R.W., Bristow M., Maisch B., Mautner B., O’Connell J., Olsen E., Thiene G., Goodwin J., Gyarfas I. (1996). Report of the 1995 World Health Organization/International Society and Federation of Cardiology Task Force on the Definition and Classification of cardiomyopathies. Circulation.

[69] O’Neill D.E., Forman D.E. (2025). Cardiomyopathy in Older Adults. Curr. Cardiol. Rep..

[70] Jin Y., Che W., Yang J., Chang S., Bao W., Ren X., Yu P., Hou A. (2025). Classification; Diagnosis, and Prognosis of Cardiomyopathy: A Comprehensive Narrative Review. Rev. Cardiovasc. Med..

[71] Qiu H., Gladysheva I.P. (2026). Therapeutic perspective on cardiomyopathy and heart failure in older adults through the lens of chronic inflammation. Vasc. Pharmacol..

[72] Brownrigg J.R.W., Leo V., Rose J., Low E., Richards S., Carr-White G., Elliott P.M. (2022). Epidemiology of cardiomyopathies and incident heart failure in a population-based cohort study. Heart.

[73] Li X., Huang A., Zhang X., Zhang Y., Zhang Z., Liu Y., Shu F., Shao S., Tan N., Jiang L. (2023). Age, GFR, and Ejection Fraction Score as a Good Predictor of Prognosis in Elderly Patients with Nonischemic Dilated Cardiomyopathy. Gerontology.

[74] Agstam S., Bahl A., Kumar R.M. (2020). Long-term outcomes of non-ischemic dilated cardiomyopathy patients with left ventricular ejection fraction ≤ 19% on medical therapy. Indian Heart J..

[75] Adwani S.S., Whitehead B.F., Rees P.G., Whitmore P., Fabre J.W., Elliott M.J., De Leval M.R. (1995). Heart transplantation for dilated cardiomyopathy. Arch. Dis. Child..

[76] Maron B.J., Spirito P., Roman M.J., Paranicas M., Okin P.M., Best L.G., Lee E.T., Devereux R.B. (2004). Prevalence of hypertrophic cardiomyopathy in a population-based sample of American Indians aged 51 to 77 years (the Strong Heart Study). Am. J. Cardiol..

[77] Maron B.J., Casey S.A., Hauser R.G., Aeppli D.M. (2003). Clinical course of hypertrophic cardiomyopathy with survival to advanced age. J. Am. Coll. Cardiol..

[78] Tsuda T., Hayashi K., Kato T., Kusayama T., Nakagawa Y., Nomura A., Tada H., Usui S., Sakata K., Kawashiri M.A. (2023). Hypertrophic Cardiomyopathy Predicts Thromboembolism and Heart Failure in Patients with Nonvalvular Atrial Fibrillation—A Prospective Analysis from the Hokuriku-Plus AF Registry. Circ. J..

[79] Sado D.M., Iqbal J. (2010). Hypertrophic cardiomyopathy in older patients. Clin. Med. J. R. Coll. Physicians Lond..

[80] Alashi A., Smedira N.G., Popovic Z.B., Fava A., Thamilarasan M., Kapadia S.R., Wierup P., Lever H.M., Desai M.Y. (2021). Characteristics and Outcomes of Elderly Patients with Hypertrophic Cardiomyopathy. J. Am. Heart Assoc..

[81] Connors L.H., Sam F., Skinner M., Salinaro F., Sun F., Ruberg F.L., Berk J.L., Seldin D.C. (2016). Heart Failure Resulting from Age-Related Cardiac Amyloid Disease Associated with Wild-Type Transthyretin: A Prospective, Observational Cohort Study. Circulation.

[82] Chan N., Teruya S., Mirabal A., Weinsaft A.Y., Santos J.D.L., Guadalupe S., Jimenez M., Rodriguez C., Helmke S., Cuomo M. (2024). Temporal Outcomes of Patients Diagnosed with Transthyretin Cardiac Amyloidosis. J. Card. Fail..

[83] Tanskanen M., Peuralinna T., Polvikoski T., Notkola I.L., Sulkava R., Hardy J., Singleton A., Kiuru-Enari S., Paetau A., Tienari P.J. (2008). Senile systemic amyloidosis affects 25% of the very aged and associates with genetic variation in alpha2-macroglobulin and tau: A population-based autopsy study. Ann. Med..

[84] Porcari A., Bussani R., Merlo M., Varrà G.G., Pagura L., Rozze D., Sinagra G. (2021). Incidence and Characterization of Concealed Cardiac Amyloidosis Among Unselected Elderly Patients Undergoing Post-mortem Examination. Front. Cardiovasc. Med..

[85] Grogan M., Scott C.G., Kyle R.A., Zeldenrust S.R., Gertz M.A., Lin G., Klarich K.W., Miller W.L., Maleszewski J.J., Dispenzieri A. (2016). Natural History of Wild-Type Transthyretin Cardiac Amyloidosis and Risk Stratification Using a Novel Staging System. J. Am. Coll. Cardiol..

[86] Zmuda L., Zaroui A., Kharoubi M., Boutin E., Roca F., Oghina S., Teiger E., Laurent M., Canoui-Poitrine F., Damy T. (2025). Tafamidis Reduces Death and Hospitalization for Acute Heart Failure in Octogenarian Patients with Transthyretin Cardiac Amyloidosis: A Propensity Score-Weighted Cohort Study. J. Am. Heart Assoc..

[87] Kubo T., Matsumura Y., Kitaoka H., Okawa M., Hirota T., Hamada T., Hitomi N., Hoshikawa E., Hayato K., Shimizu Y. (2008). Improvement in prognosis of dilated cardiomyopathy in the elderly over the past 20 years. J. Cardiol..

[88] Olivotto I., Oreziak A., Barriales-Villa R., Abraham T.P., Masri A., Garcia-Pavia P., Saberi S., Lakdawala N.K., Wheeler M.T., Owens A. (2020). Mavacamten for treatment of symptomatic obstructive hypertrophic cardiomyopathy (EXPLORER-HCM): A randomised, double-blind, placebo-controlled, phase 3 trial. Lancet.

[89] Jenča D., Melenovský V., Stehlik J., Staněk V., Kettner J., Kautzner J., Adámková V., Wohlfahrt P. (2021). Heart failure after myocardial infarction: Incidence and predictors. ESC Heart Fail..

[90] Chen L., Li S., Zhu J., You A., Huang X., Yi X., Xue M. (2021). Mangiferin prevents myocardial infarction-induced apoptosis and heart failure in mice by activating the Sirt1/FoxO3a pathway. J. Cell. Mol. Med..

[91] Zhao K., Xu T., Mao Y., Wu X., Hua D., Sheng Y., Li P. (2022). Alamandine alleviated heart failure and fibrosis in myocardial infarction mice. Biol. Direct.

[92] Almesned M.A., van Essen B.J., Emmens J.E., Tromp J., Smit M.D., Geluk C.A., Gansevoort R.T., Bakker S.J.L., Damman K., de Boer R.A. (2025). Association between antecedent myocardial infarction and heart failure with preserved versus reduced ejection fraction. Eur. J. Heart Fail..

[93] Ennezat P.V., Lamblin N., Mouquet F., Tricot O., Quandalle P., Aumégeat V., Equine O., Nugue O., Segrestin B., De Groote P. (2008). The effect of ageing on cardiac remodelling and hospitalization for heart failure after an inaugural anterior myocardial infarction. Eur. Heart J..

[94] Ezekowitz J.A., Kaul P., Bakal J.A., Armstrong P.W., Welsh R.C., McAlister F.A. (2009). Declining in-hospital mortality and increasing heart failure incidence in elderly patients with first myocardial infarction. J. Am. Coll. Cardiol..

[95] Sulo G., Igland J., Vollset S.E., Nygård O., Ebbing M., Sulo E., Egeland G.M., Tell G.S. (2016). Heart Failure Complicating Acute Myocardial Infarction; Burden and Timing of Occurrence: A Nation-wide Analysis Including 86 771 Patients from the Cardiovascular Disease in Norway (CVDNOR) Project. J. Am. Heart Assoc..

[96] Shah R.V., Holmes D.J., Anderson M., Wang T.Y., Kontos M.C., Wiviott S.D., Scirica B.M. (2012). Risk of heart failure complication during hospitalization for acute myocardial infarction in a contemporary population: Insights from the National Cardiovascular Data ACTION Registry. Circ. Heart Fail..

[97] Leening M.J.G., Elias-Smale S.E., Felix J.F., Kors J.A., Deckers J.W., Hofman A., Stricker B.H.C., Witteman J.C.M. (2010). Unrecognised myocardial infarction and long-term risk of heart failure in the elderly: The Rotterdam Study. Heart.

[98] Nishihira K., Kuriyama N., Kadooka K., Honda Y., Yamamoto K., Nishino S., Ebihara S., Ogata K., Kimura T., Koiwaya H. (2022). Outcomes of Elderly Patients with Acute Myocardial Infarction and Heart Failure Who Undergo Percutaneous Coronary Intervention. Circ. Rep..

[99] Yin X., Huang C., Lin B. (2024). Application of intensive management of risk awareness combined with cardiac rehabilitation nursing in elderly patients with acute myocardial infarction and heart failure. Medicine.

[100] Kwiecinski J., Lennen R.J., Gray G.A., Borthwick G., Boswell L., Baker A.H., Newby D.E., Dweck M.R., Jansen M.A. (2020). Progression and regression of left ventricular hypertrophy and myocardial fibrosis in a mouse model of hypertension and concomitant cardiomyopathy. J. Cardiovasc. Magn. Reson..

[101] Raman S.V. (2010). The Hypertensive Heart: An Integrated Understanding Informed by Imaging. J. Am. Coll. Cardiol..

[102] Leache L., Gutiérrez-Valencia M., Finizola R.M., Infante E., Finizola B., Pardo J.P., Flores Y., Granero R., Arai K.J. (2021). Pharmacotherapy for hypertension-induced left ventricular hypertrophy. Cochrane Database Syst. Rev..

[103] Zhang H., Singal P.K., Ravandi A., Rabinovich-Nikitin I. (2025). Sex-Specific Differences in the Pathophysiology of Hypertension. Biomolecules.

[104] Gallo G., Savoia C. (2024). Hypertension and Heart Failure: From Pathophysiology to Treatment. Int. J. Mol. Sci..

[105] Sahle B.W., Owen A.J., Krum H., Reid C.M. (2016). Incidence of heart failure in 6083 elderly hypertensive patients: The Second Australian National Blood Pressure Study (ANBP2). Eur. J. Heart Fail..

[106] Butler J., Kalogeropoulos A.P., Georgiopoulou V.V., Bibbins-Domingo K., Najjar S.S., Sutton-Tyrrell K.C., Harris T.B., Kritchevsky S.B., Lloyd-Jones D.M., Newman A.B. (2011). Systolic blood pressure and incident heart failure in the elderly. The Cardiovascular Health Study and the Health, Ageing and Body Composition Study. Heart.

[107] Putot S., Hacquin A., Manckoundia P., Putot A. (2021). Prognostic impact of systolic blood pressure in acute heart failure with preserved ejection fraction in older patients. ESC Heart Fail..

[108] Yang W., Zhou Y.J., Fu Y., Qin J., Tan S., Chen X.M., Guo J.C., Wang D.Z., Zhan H., Guan W. (2016). Therapeutic effects of intravenous urapidil in elderly patients with hypertension and acute decompensated heart failure: A pilot clinical trial. Exp. Ther. Med..

[109] Li X., Zuo C., Chen C., Tian D., Li J., Fan L., Li X., Lv Q. (2023). Effectiveness and safety evaluation of sacubitril/valsartan in blood pressure control and clinical outcomes for elderly patients with heart failure and hypertension: A prospective cohort study. Int. J. Cardiol..

[110] Chen L., Qian L., Liu Y. (2024). Association Between Different Insulin Resistance Indices and Heart Failure in US Adults with Diabetes Mellitus. Ann. Noninvasive Electrocardiol..

[111] Peng C., Zhang Y., Lang X., Zhang Y. (2023). Role of mitochondrial metabolic disorder and immune infiltration in diabetic cardiomyopathy: New insights from bioinformatics analysis. J. Transl. Med..

[112] Hoek A.G., Canto E.D., Wenker E., Bindraban N., Handoko M.L., Elders P.J.M., Beulens J.W.J. (2024). Epidemiology of heart failure in diabetes: A disease in disguise. Diabetologia.

[113] Boonman-De Winter L.J.M., Rutten F.H., Cramer M.J.M., Landman M.J., Liem A.H., Rutten G.E.H.M., Hoes A.W. (2012). High prevalence of previously unknown heart failure and left ventricular dysfunction in patients with type 2 diabetes. Diabetologia.

[114] Echouffo-Tcheugui J.B., Xu H., DeVore A.D., Schulte P.J., Butler J., Yancy C.W., Bhatt D.L., Hernandez A.F., Heidenreich P.A., Fonarow G.C. (2016). Temporal trends and factors associated with diabetes mellitus among patients hospitalized with heart failure: Findings from Get with The Guidelines-Heart Failure registry. Am. Heart J..

[115] Eguchi K., Boden-Albala B., Jin Z., Rundek T., Sacco R.L., Homma S., Di Tullio M.R. (2008). Association Between Diabetes Mellitus and Left Ventricular Hypertrophy in a Multi-Ethnic Population. Am. J. Cardiol..

[116] Wong T.C., Piehler K.M., Kang I.A., Kadakkal A., Kellman P., Schwartzman D.S., Mulukutla S.R., Simon M.A., Shroff S.G., Kuller L.H. (2014). Myocardial extracellular volume fraction quantified by cardiovascular magnetic resonance is increased in diabetes and associated with mortality and incident heart failure admission. Eur. Heart J..

[117] Levelt E., Mahmod M., Piechnik S.K., Ariga R., Francis J.M., Rodgers C.T., Clarke W.T., Sabharwal N., Schneider J.E., Karamitsos T.D. (2016). Relationship Between Left Ventricular Structural and Metabolic Remodeling in Type 2 Diabetes. Diabetes.

[118] Wang Y., Yang H., Huynh Q., Nolan M., Negishi K., Marwick T.H. (2018). Diagnosis of Nonischemic Stage B Heart Failure in Type 2 Diabetes Mellitus: Optimal Parameters for Prediction of Heart Failure, JACC Cardiovasc. Imaging.

[119] Rosengren A., Vestberg D., Svensson A.M., Kosiborod M., Clements M., Rawshani A., Pivodic A., Gudbjörnsdottir S., Lind M. (2015). Long-term excess risk of heart failure in people with type 1 diabetes: A prospective case-control study. Lancet Diabetes Endocrinol..

[120] Segar M.W., Patel K.V., Vaduganathan M., Caughey M.C., Butler J., Fonarow G.C., Grodin J.L., McGuire D.K., Pandey A. (2020). Association of Long-term Change and Variability in Glycemia with Risk of Incident Heart Failure Among Patients with Type 2 Diabetes: A Secondary Analysis of the ACCORD Trial. Diabetes Care.

[121] Deedwania P., Patel K., Fonarow G.C., Desai R.V., Zhang Y., Feller M.A., Ovalle F., Love T.E., Aban I.B., Mujib M. (2013). Prediabetes is not an independent risk factor for incident heart failure, other cardiovascular events or mortality in older adults: Findings from a population-based cohort study. Int. J. Cardiol..

[122] Kato E.T., Silverman M.G., Mosenzon O., Zelniker T.A., Cahn A., Furtado R.H.M., Kuder J., Murphy S.A., Bhatt D.L., Leiter L.A. (2019). Effect of Dapagliflozin on Heart Failure and Mortality in Type 2 Diabetes Mellitus. Circulation.

[123] Kenchaiah S., Sesso H.D., Gaziano J.M. (2009). Body mass index and vigorous physical activity and the risk of heart failure among men. Circulation.

[124] Packer M., Zile M.R., Kramer C.M., Baum S.J., Litwin S.E., Menon V., Ge J., Weerakkody G.J., Ou Y., Bunck M.C. (2025). Tirzepatide for Heart Failure with Preserved Ejection Fraction and Obesity. N. Engl. J. Med..

[125] Cai A., Liu L., Zhou D., Tang S., Tadic M., Schutte A.E., Feng Y. (2024). Obesity and Risk of Incident Left Ventricular Hypertrophy in Community-Dwelling Populations with Hypertension: An Observational Study. J. Am. Heart Assoc..

[126] Cobos-Palacios L., Ruiz-Moreno M.I., Vilches-Perez A., Vargas-Candela A., Muñoz-Úbeda M., Porres J.B., Navarro-Sanz A., Lopez-Carmona M.D., Sanz-Canovas J., Perez-Belmonte L.M. (2022). Metabolically healthy obesity: Inflammatory biomarkers and adipokines in elderly population. PLoS ONE.

[127] Savji N., Meijers W.C., Bartz T.M., Bhambhani V., Cushman M., Nayor M., Kizer J.R., Sarma A., Blaha M.J., Gansevoort R.T. (2018). The Association of Obesity and Cardiometabolic Traits with Incident HFpEF and HFrEF. JACC Heart Fail..

[128] Russo C., Jin Z., Homma S., Rundek T., Elkind M.S.V., Sacco R.L., Di Tullio M.R. (2011). Effect of obesity and overweight on left ventricular diastolic function: A community-based study in an elderly cohort. J. Am. Coll. Cardiol..

[129] Murphy N.F., MacIntyre K., Stewart S., Hart C.L., Hole D., McMurray J.J.V. (2006). Long-term cardiovascular consequences of obesity: 20-year follow-up of more than 15 000 middle-aged men and women (the Renfrew-Paisley study). Eur. Heart J..

[130] Piepoli M.F., Corrà U., Veglia F., Bonomi A., Salvioni E., Cattadori G., Metra M., Lombardi C., Sinagra G., Limongelli G. (2016). Exercise tolerance can explain the obesity paradox in patients with systolic heart failure: Data from the MECKI Score Research Group. Eur. J. Heart Fail..

[131] Testa G., Cacciatore F., Galizia G., Della-Morte D., Mazzella F., Langellotto A., Russo S., Gargiulo G., De Santis D., Ferrara N. (2010). Waist circumference but not body mass index predicts long-term mortality in elderly subjects with chronic heart failure. J. Am. Geriatr. Soc..

[132] Kitzman D.W., Brubaker P., Morgan T., Haykowsky M., Hundley G., Kraus W.E., Eggebeen J., Nicklas B.J. (2016). Effect of Caloric Restriction or Aerobic Exercise Training on Peak Oxygen Consumption and Quality of Life in Obese Older Patients with Heart Failure with Preserved Ejection Fraction: A Randomized Clinical Trial. JAMA.

[133] Brubaker P.H., Nicklas B.J., Houston D.K., Hundley W.G., Chen H., Molina A.J.A., Lyles W.M., Nelson B., Upadhya B., Newland R. (2023). Controlled Trial of Resistance Training Added to Caloric Restriction Plus Aerobic Exercise Training in Obese Heart Failure with Preserved Ejection Fraction. Circ. Heart Fail..

[134] Haykowsky M.J., Nicklas B.J., Brubaker P.H., Hundley W.G., Brinkley T.E., Upadhya B., Becton J.T., Nelson M.D., Chen H., Kitzman D.W. (2018). Regional Adipose Distribution and its Relationship to Exercise Intolerance in Older Obese Patients Who Have Heart Failure with Preserved Ejection Fraction. JACC Heart Fail.

[135] Sundström J., Bruze G., Ottosson J., Marcus C., Näslund I., Neovius M. (2017). Weight Loss and Heart Failure: A Nationwide Study of Gastric Bypass Surgery Versus Intensive Lifestyle Treatment. Circulation.

[136] Chieng D., Sugumar H., Segan L., Tan C., Vizi D., Nanayakkara S., Al-Kaisey A., Hawson J., Prabhu S., Voskoboinik A. (2023). Atrial Fibrillation Ablation for Heart Failure with Preserved Ejection Fraction: A Randomized Controlled Trial. JACC Heart Fail..

[137] Reddy Y.N.V., Obokata M., Verbrugge F.H., Lin G., Borlaug B.A. (2020). Atrial Dysfunction in Patients with Heart Failure with Preserved Ejection Fraction and Atrial Fibrillation. J. Am. Coll. Cardiol..

[138] Haller P.M., Jarolim P., Palazzolo M.G., Bellavia A., Antman E.M., Eikelboom J., Granger C.B., Harrington J., Healey J.S., Hijazi Z. (2024). Heart Failure Risk Assessment Using Biomarkers in Patients with Atrial Fibrillation: Analysis from COMBINE-AF. J. Am. Coll. Cardiol..

[139] Santhanakrishnan R., Wang N., Larson M.G., Magnani J.W., McManus D.D., Lubitz S.A., Ellinor P.T., Cheng S., Vasan R.S., Lee D.S. (2016). Atrial Fibrillation Begets Heart Failure and Vice Versa: Temporal Associations and Differences in Preserved Versus Reduced Ejection Fraction. Circulation.

[140] Alenazy B., Tharkar S., Kashour T., Alhabib K.F., Alfaleh H., Hersi A. (2019). In-hospital ventricular arrhythmia in heart failure patients: 7 year follow-up of the multi-centric HEARTS registry. ESC Heart Fail..

[141] Brachmann J., Sohns C., Andresen D., Siebels J., Sehner S., Boersma L., Merkely B., Pokushalov E., Sanders P., Schunkert H. (2021). Atrial Fibrillation Burden and Clinical Outcomes in Heart Failure: The CASTLE-AF Trial. JACC Clin. Electrophysiol..

[142] Verma S., Butler J., Borlaug B.A., Davies M.J., Kitzman D.W., Petrie M.C., Shah S.J., Jensen T.J., Rasmussen S., Rönnbäck C. (2024). Atrial Fibrillation and Semaglutide Effects in Obesity-Related Heart Failure with Preserved Ejection Fraction: STEP-HFpEF Program. J. Am. Coll. Cardiol..

[143] Mehta R., Ning H., Bansal N., Cohen J., Srivastava A., Dobre M., Michos E.D., Rahman M., Townsend R., Seliger S. (2022). Ten-Year Risk-Prediction Equations for Incident Heart Failure Hospitalizations in Chronic Kidney Disease: Findings from the Chronic Renal Insufficiency Cohort Study and the Multi-Ethnic Study of Atherosclerosis. J. Card. Fail..

[144] Patel S.S., Raman V.K., Zhang S., Deedwania P., Zeng-Treitler Q., Wu W.C., Lam P.H., Bakris G., Moore H., Heidenreich P.A. (2024). Identification and outcomes of KDIGO-defined chronic kidney disease in 1.4 million U.S. Veterans with heart failure. Eur. J. Heart Fail..

[145] Patel R.N., Sharma A., Prasad A., Bansal S. (2023). Heart Failure with Preserved Ejection Fraction with CKD: A Narrative Review of a Multispecialty Disorder. Kidney Med..

[146] Kirkman D.L., Carbone S., Canada J.M., Trankle C., Kadariya D., Buckley L., Billingsley H., Kidd J.M., Van Tassell B.W., Abbate A. (2021). The Chronic Kidney Disease Phenotype of HFpEF: Unique Cardiac Characteristics. Am. J. Cardiol..

[147] Buckley L.F., Claggett B.L., Matsushita K., McMahon G.M., Skali H., Coresh J., Folsom A.R., Konety S.H., Wagenknecht L.E., Mosley T.H. (2023). Chronic Kidney Disease, Heart Failure, and Adverse Cardiac Remodeling in Older Adults: The ARIC Study. JACC Heart Fail..

[148] Bansal N., Zelnick L., Bhat Z., Dobre M., He J., Lash J., Jaar B., Mehta R., Raj D., Rincon-Choles H. (2019). Burden and Outcomes of Heart Failure Hospitalizations in Adults with Chronic Kidney Disease. J. Am. Coll. Cardiol..

[149] Pratley R.E., Tuttle K.R., Rossing P., Rasmussen S., Perkovic V., Nielsen O.W., Mann J.F.E., MacIsaac R.J., Kosiborod M.N., Kamenov Z. (2024). Effects of Semaglutide on Heart Failure Outcomes in Diabetes and Chronic Kidney Disease in the FLOW Trial. J. Am. Coll. Cardiol..

[150] Filippatos G., Anker S.D., Pitt B., Rossing P., Joseph A., Kolkhof P., Lambelet M., Lawatscheck R., Bakris G.L., Ruilope L.M. (2022). Finerenone and Heart Failure Outcomes by Kidney Function/Albuminuria in Chronic Kidney Disease and Diabetes. JACC Heart Fail..

[151] Packer M., Zile M.R., Kramer C.M., Murakami M., Ou Y., Borlaug B.A. (2025). Interplay of Chronic Kidney Disease and the Effects of Tirzepatide in Patients with Heart Failure, Preserved Ejection Fraction, and Obesity: The SUMMIT Trial. J. Am. Coll. Cardiol..

[152] Tavazzi L., Swedberg K., Komajda M., Böhm M., Borer J.S., Lainscak M., Robertson M., Ford I. (2013). Clinical profiles and outcomes in patients with chronic heart failure and chronic obstructive pulmonary disease: An efficacy and safety analysis of SHIFT study. Int. J. Cardiol..

[153] Hesse K., Bourke S., Steer J. (2022). Heart failure in patients with COPD exacerbations: Looking below the tip of the iceberg. Respir. Med..

[154] Alvarez-Martinez C.J., Hernández M., Vélez J., Sánchez-Covisa J., Moreno-González J., Escudero L., Rosillo N., Alvaredo D., Moreno G., del Oro M. (2025). Clinical and economic impact of concomitant heart failure in patients with exacerbated COPD. Respir. Med..

[155] Xu S., Gu Z., Zhu W., Feng S. (2025). Association of COPD with adverse outcomes in heart failure patients with preserved ejection fraction. ESC Heart Fail..

[156] Mooney L., Hawkins N.M., Jhund P.S., Redfield M.M., Vaduganathan M., Desai A.S., Rouleau J.L., Minamisawa M., Shah A.M., Lefkowitz M.P. (2021). Impact of Chronic Obstructive Pulmonary Disease in Patients with Heart Failure with Preserved Ejection Fraction: Insights from PARAGON-HF. J. Am. Heart Assoc..

[157] Butt J.H., Lu H., Kondo T., Bachus E., de Boer R.A., Inzucchi S.E., Jhund P.S., Kosiborod M.N., Lam C.S.P., Martinez F.A. (2023). Heart failure, chronic obstructive pulmonary disease and efficacy and safety of dapagliflozin in heart failure with mildly reduced or preserved ejection fraction: Insights from DELIVER. Eur. J. Heart Fail..

[158] Dewan P., Docherty K.F., Bengtsson O., de Boer R.A., Desai A.S., Drozdz J., Hawkins N.M., Inzucchi S.E., Kitakaze M., Køber L. (2021). Effects of dapagliflozin in heart failure with reduced ejection fraction and chronic obstructive pulmonary disease: An analysis of DAPA-HF. Eur. J. Heart Fail..

[159] Beer B.N., Benson L., Basile C., Schrage B., Becher P.M., Blankenberg S., Kirchhof P., Szabó-Söderberg B., Metra M., Lindberg A. (2025). Beta-blockers in patients with heart failure with reduced ejection fraction and concomitant chronic obstructive pulmonary disease: Cardiovascular and respiratory outcomes. Eur. J. Heart Fail..

[160] Huang S., Cai T., Weber B.N., He Z., Dahal K.P., Hong C., Hou J., Seyok T., Cagan A., DiCarli M.F. (2023). Association Between Inflammation, Incident Heart Failure, and Heart Failure Subtypes in Patients with Rheumatoid Arthritis. Arthritis Care Res..

[161] Ahlers M.J., Lowery B.D., Farber-Eger E., Wang T.J., Bradham W., Ormseth M.J., Chung C.P., Stein C.M., Gupta D.K. (2020). Heart Failure Risk Associated with Rheumatoid Arthritis-Related Chronic Inflammation. J. Am. Heart Assoc..

[162] Kalogeropoulos A., Georgiopoulou V., Psaty B.M., Rodondi N., Smith A.L., Harrison D.G., Liu Y., Hoffmann U., Bauer D.C., Newman A.B. (2010). Inflammatory Markers and Incident Heart Failure Risk in Older Adults. The Health ABC (Health, Aging, and Body Composition) Study. J. Am. Coll. Cardiol..

[163] Zheng H., Yin Z., Luo X., Zhou Y., Zhang F., Guo Z. (2024). Associations between systemic immunity-inflammation index and heart failure: Evidence from the NHANES 1999–2018. Int. J. Cardiol..

[164] Michou E., Wussler D., Belkin M., Simmen C., Strebel I., Nowak A., Kozhuharov N., Shrestha S., Lopez-Ayala P., Sabti Z. (2023). Quantifying inflammation using interleukin-6 for improved phenotyping and risk stratification in acute heart failure. Eur. J. Heart Fail..

[165] Hanna A., Frangogiannis N.G. (2020). Inflammatory Cytokines and Chemokines as Therapeutic Targets in Heart Failure. Cardiovasc. Drugs Ther..

[166] Everett B.M., Cornel J.H., Lainscak M., Anker S.D., Abbate A., Thuren T., Libby P., Glynn R.J., Ridker P.M. (2019). Anti-Inflammatory Therapy with Canakinumab for the Prevention of Hospitalization for Heart Failure. Circulation.

[167] Buckley L.F., Carbone S., Trankle C.R., Canada J.M., Erdle C.O., Regan J.A., Viscusi M.M., Kadariya D., Billingsley H., Arena R. (2018). Effect of Interleukin-1 Blockade on Left Ventricular Systolic Performance and Work: A Post Hoc Pooled Analysis of 2 Clinical Trials. J. Cardiovasc. Pharmacol..

[168] Yamashita S., Saotome M., Saitoh T., Ogawa N., Maekawa Y. (2022). Long-term effect of tocilizumab on left ventricular hypertrophy and systolic dysfunction in AA amyloidosis with rheumatoid arthritis. J. Cardiol. Cases.

[169] Friedman S.L., Ratziu V., Harrison S.A., Abdelmalek M.F., Aithal G.P., Caballeria J., Francque S., Farrell G., Kowdley K.V., Craxi A. (2018). A randomized, placebo-controlled trial of cenicriviroc for treatment of nonalcoholic steatohepatitis with fibrosis. Hepatology.

[170] Papamichail A., Kourek C., Briasoulis A., Xanthopoulos A., Tsougos E., Farmakis D., Paraskevaidis I. (2023). Targeting Key Inflammatory Mechanisms Underlying Heart Failure: A Comprehensive Review. Int. J. Mol. Sci..

[171] Berger M., März W., Niessner A., Delgado G., Kleber M., Scharnagl H., Marx N., Schuett K. (2024). IL-6 and hsCRP predict cardiovascular mortality in patients with heart failure with preserved ejection fraction. ESC Heart Fail..

[172] Mooney L., Jackson C.E., Adamson C., McConnachie A., Welsh P., Myles R.C., McMurray J.J.V., Jhund P.S., Petrie M.C., Lang N.N. (2023). Adverse Outcomes Associated with Interleukin-6 in Patients Recently Hospitalized for Heart Failure with Preserved Ejection Fraction. Circ. Heart Fail..

[173] Hage C., Michaëlsson E., Linde C., Donal E., Daubert J.C., Gan L.M., Lund L.H. (2017). Inflammatory Biomarkers Predict Heart Failure Severity and Prognosis in Patients with Heart Failure with Preserved Ejection Fraction: A Holistic Proteomic Approach. Circ. Cardiovasc. Genet..

[174] Sharma A., Stevens S.R., Lucas J., Fiuzat M., Adams K.F., Whellan D.J., Donahue M.P., Kitzman D.W., Piña I.L., Zannad F. (2017). Utility of Growth Differentiation Factor-15, A Marker of Oxidative Stress and Inflammation, in Chronic Heart Failure: Insights from the HF-ACTION Study. JACC Heart Fail..

[175] Wang Z., Qin Z., Yuan R., Guo J., Xu S., Lv Y., Xu Y., Lu Y., Gao J., Yu F. (2023). Systemic immune-inflammation index as a prognostic marker for advanced chronic heart failure with renal dysfunction. ESC Heart Fail..

[176] Nagasaka R., Kim E., Ambrosy A.P., Feinstein M.J. (2025). Targeting inflammation in heart failure: Evolving insights and future directions from randomized clinical trials. Heart Fail. Rev..

[177] Centner A.M., Cullen A.E., Khalili L., Ukhanov V., Hill S., Deitado R., Hwang H.S., Azeez T., La Favor J.D., Laitano O. (2025). The Role of Sex in the Effects of Smoking and Nicotine on Cardiovascular Function, Atherosclerosis, and Inflammation. Nicotine Tob. Res..

[178] Hunt L.J., Covinsky K.E., Cenzer I., Espejo E., Boscardin W.J., Leutwyler H., Lee A.K., Cataldo J. (2023). The Epidemiology of Smoking in Older Adults: A National Cohort Study. J. Gen. Intern. Med..

[179] Gopal D.M., Kalogeropoulos A.P., Georgiopoulou V.V., Smith A.L., Bauer D.C., Newman A.B., Kim L., Bibbins-Domingo K., Tindle H., Harris T.B. (2012). Cigarette Smoking Exposure and Heart Failure Risk in Older Adults: The Health, Aging, and Body Composition Study. Am. Heart J..

[180] Sandesara P.B., Samman-Tahhan A., Topel M., Venkatesh S., O’Neal W.T. (2018). Effect of Cigarette Smoking on Risk for Adverse Events in Patients with Heart Failure and Preserved Ejection Fraction. Am. J. Cardiol..

[181] Feodoroff M., Harjutsalo V., Forsblom C., Groop P.H. (2018). Dose-dependent effect of smoking on risk of coronary heart disease, heart failure and stroke in individuals with type 1 diabetes. Diabetologia.

[182] Kamimura D., Cain L.R., Mentz R.J., White W.B., Blaha M.J., Defilippis A.P., Fox E.R., Rodriguez C.J., Keith R.J., Benjamin E.J. (2018). Cigarette Smoking and Incident Heart Failure: Insights from the Jackson Heart Study. Circulation.

[183] Ahmed A.A., Patel K., Nyaku M.A., Kheirbek R.E., Bittner V., Fonarow G.C., Filippatos G.S., Morgan C.J., Aban I.B., Mujib M. (2015). Risk of Heart Failure and Death After Prolonged Smoking Cessation: Role of Amount and Duration of Prior Smoking. Circ. Heart Fail..

[184] Gottdiener J.S., Buzkova P., Kahn P.A., DeFilippi C., Shah S., Barasch E., Kizer J.R., Psaty B., Gardin J.M. (2022). Relation of Cigarette Smoking and Heart Failure in Adults ≥ 65 Years of Age (from the Cardiovascular Health Study). Am. J. Cardiol..

[185] Ding N., Shah A.M., Blaha M.J., Chang P.P., Rosamond W.D., Matsushita K. (2022). Cigarette Smoking, Cessation, and Risk of Heart Failure with Preserved and Reduced Ejection Fraction. J. Am. Coll. Cardiol..

[186] Polecka A., Olszewska N., Danielski Ł., Olszewska E. (2023). Association between Obstructive Sleep Apnea and Heart Failure in Adults-A Systematic Review. J. Clin. Med..

[187] Khayat R.N., Javaheri S., Porter K., Sow A., Holt R., Randerath W., Abraham W.T., Jarjoura D. (2020). In-Hospital Management of Sleep Apnea During Heart Failure Hospitalization: A Randomized Controlled Trial. J. Card. Fail..

[188] Khayat R., Jarjoura D., Porter K., Sow A., Wannemacher J., Dohar R., Pleister A., Abraham W.T. (2015). Sleep disordered breathing and post-discharge mortality in patients with acute heart failure. Eur. Heart J..

[189] Sommerfeld A., Althouse A.D., Prince J., Atwood C.W., Mulukutla S.R., Hickey G.W. (2017). Obstructive sleep apnea is associated with increased readmission in heart failure patients. Clin. Cardiol..

[190] Larsson S.C., Burgess S., Mason A.M., Michaëlsson K. (2020). Alcohol Consumption and Cardiovascular Disease: A Mendelian Randomization Study. Circ. Genom. Precis. Med..

[191] Yeo Y., Jeong S.M., Shin D.W., Han K., Yoo J., Yoo J.E., Lee S.P. (2022). Changes in Alcohol Consumption and Risk of Heart Failure: A Nationwide Population-Based Study in Korea. Int. J. Environ. Res. Public Health.

[192] Nguyen X.M.T., Elhouderi E., Li Y., Williams A.R., Gaziano L., Joseph J., Gaziano J.M., Cho K., Djousse L. (2026). Alcohol Intake and Incidence of Heart Failure and Its Subtypes: VA Million Veteran Program. Nutrients.

[193] Klatsky A.L., Chartier D., Udaltsova N., Gronningen S., Brar S., Friedman G.D., Lundstrom R.J. (2005). Alcohol drinking and risk of hospitalization for heart failure with and without associated coronary artery disease. Am. J. Cardiol..

[194] Abramson J.L., Williams S.A., Krumholz H.M., Vaccarino V. (2001). Moderate alcohol consumption and risk of heart failure among older persons. JAMA.

[195] Walsh C.R., Larson M.G., Evans J.C., Djousse L., Ellison R.C., Vasan R.S., Levy D. (2002). Alcohol consumption and risk for congestive heart failure in the Framingham Heart Study. Ann. Intern. Med..

[196] Heidenreich P.A., Albert N.M., Allen L.A., Bluemke D.A., Butler J., Fonarow G.C., Ikonomidis J.S., Khavjou O., Konstam M.A., Maddox T.M. (2013). Forecasting the Impact of Heart Failure in the United States: A Policy Statement from the American Heart Association. Circ. Heart Fail..

[197] Voors A.A., Angermann C.E., Teerlink J.R., Collins S.P., Kosiborod M., Biegus J., Ferreira J.P., Nassif M.E., Psotka M.A., Tromp J. (2022). The SGLT2 inhibitor empagliflozin in patients hospitalized for acute heart failure: A multinational randomized trial. Nat. Med..

[198] Yusuf S., Pfeffer M.A., Swedberg K., Granger C.B., Held P., McMurray J.J.V., Michelson E.L., Olofsson B., Östergren J. (2003). Effects of candesartan in patients with chronic heart failure and preserved left-ventricular ejection fraction: The CHARM-Preserved Trial. Lancet.

