# Correction: Flores et al. The Impact of Risk Factors and Comorbidities on Heart Failure in the Aging Population. *Int. J. Mol. Sci.* 2026, *27*, 5296

**DOI:** 10.3390/ijms27146160

**Published:** 2026-07-10

**Authors:** Ruzzell C. Flores, Fatin Shadab Talukder, Inna Rabinovich-Nikitin

**Affiliations:** 1 Department of Physiology and Pathophysiology, Rady College of Medicine, Max Rady Faculty of Health Sciences, University of Manitoba, Winnipeg, MB R2H2A6, Canada; rflores@sbrc.ca (R.C.F.);; 2 The Institute of Cardiovascular Sciences, St. Boniface Hospital Albrechtsen Research Centre, Winnipeg, MB R2H2A6, Canada

**Replacement Reference(s)**

In the published article [1], Reference 150, originally cited as Butt et al., has been corrected to the intended reference, Filippatos et al. (2022). The corresponding in-text citation number remains unchanged. The authors state that the scientific conclusions are unaffected. This correction was approved by the Academic Editor. The original publication has also been updated. 

Original ref. [150]:

150. Butt, J.H.; Jhund, P.S.; Henderson, A.D.; Claggett, B.L.; Desai, A.S.; Lam, C.S.P.; Mueller, K.; Scheerer, M.F.; Viswanathan, P.; Senni, M.; et al. Finerenone, chronic obstructive pulmonary disease, and heart failure with mildly reduced or preserved ejection fraction: A prespecified analysis of the FINEARTS-HF trial. *Eur. J. Heart Fail.* **2025**, *27*, 1444–1458. https://doi.org/10.1002/EJHF.3661.

Corrected ref. [150]:

150. Filippatos, G.; Anker, S.D.; Pitt, B.; Rossing, P.; Joseph, A.; Kolkhof, P.; Lambelet, M.; Lawatscheck, R.; Bakris, G.L.; Ruilope, L.M.; et al. Finerenone and Heart Failure Outcomes by Kidney Function/Albuminuria in Chronic Kidney Disease and Diabetes. *JACC Heart Fail.* **2022**, *10*, 860–870. https://doi.org/10.1016/j.jchf.2022.07.013. Erratum in *JACC Heart Fail.* **2023**, *11*, 1034–1035. https://doi.org/10.1016/j.jchf.2023.06.002.
